# The landscape of multiscale transcriptomic networks and key regulators in Parkinson’s disease

**DOI:** 10.1038/s41467-019-13144-y

**Published:** 2019-11-20

**Authors:** Qian Wang, Yuanxi Zhang, Minghui Wang, Won-Min Song, Qi Shen, Andrew McKenzie, Insup Choi, Xianxiao Zhou, Ping-Yue Pan, Zhenyu Yue, Bin Zhang

**Affiliations:** 1 Department of Neurology and Neuroscience, Friedman Brain Institute, Icahn School of Medicine at Mount Sinai, 1425 Madison Avenue, New York, NY 10029 USA; 2 Department of Genetics and Genomic Sciences, Icahn School of Medicine at Mount Sinai, 1425 Madison Avenue, New York, NY 10029 USA; 3 Mount Sinai Center for Transformative Disease Modeling, Icahn School of Medicine at Mount Sinai, 1470 Madison Avenue, New York, NY 10029 USA; 4 Icahn Institute of Genomics and Multi-scale Biology, Icahn School of Medicine at Mount Sinai, 1425 Madison Avenue, New York, NY 10029 USA

**Keywords:** Transcriptomics, Synaptic transmission, Regulatory networks, Parkinson's disease

## Abstract

Genetic and genomic studies have advanced our knowledge of inherited Parkinson’s disease (PD), however, the etiology and pathophysiology of idiopathic PD remain unclear. Herein, we perform a meta-analysis of 8 PD postmortem brain transcriptome studies by employing a multiscale network biology approach to delineate the gene-gene regulatory structures in the substantia nigra and determine key regulators of the PD transcriptomic networks. We identify *STMN2*, which encodes a stathmin family protein and is down-regulated in PD brains, as a key regulator functionally connected to known PD risk genes. Our network analysis predicts a function of human *STMN2* in synaptic trafficking, which is validated in *Stmn2*-knockdown mouse dopaminergic neurons. *Stmn2* reduction in the mouse midbrain causes dopaminergic neuron degeneration, phosphorylated α-synuclein elevation, and locomotor deficits. Our integrative analysis not only begins to elucidate the global landscape of PD transcriptomic networks but also pinpoints potential key regulators of PD pathogenic pathways.

## Abstract (web summary)

Parkinson’s disease (PD) is characterized by neurodegeneration associated with loss of dopaminergic (DA) neurons and deposition of Lewy bodies. Here, Wang et al. use co-expression network analysis to pinpoint disease pathways and propose reduced expression of STMN2 as a cause of presynaptic function loss in PD.

## Introduction

Parkinson’s disease (PD) is a common neurodegenerative disorder characterized pathologically by the loss of dopaminergic (DA, or tyrosine hydroxylase positive, TH+) neurons in the substantia nigra (SN) and the presence of Lewy bodies and Lewy neurites in affected brain regions. Previous research has identified over 20 PD causal mutations in *SNCA, LRRK2, VPS35, PINK1, DJ-1, Parkin, FBXO7, DNAJC6, ATP13A2, DCTN1*, and *SYNJ1* (for review, see ref. ^1^ and MDS Taskforce database: www.mdsgene.org). Tremendous effort has been made to characterize the functions of these genes. For example, *SNCA* has been reported to be localized at presynapse through lipid-raft binding^2^ and play a role in vesicular trafficking^3^. *LRRK2* has been implicated in a variety of cellular processes, including autophagy^4^, mitochondrial function^5^, and vesicular trafficking^6^. *VPS35* is a core component of the retromer complex responsible for the retrograde transport of proteins in endosomes to the trans-Golgi network^7^. These PD genes are involved in multiple cellular pathways, including ubiquitin–proteasome degradations, chaperone activities and endosomal–lysosomal dynamics, as well as mitochondrial maintenance and mitophagy^1^.

However, genetic variants account for ~20% of PD cases^8^, while the etiology of the majority or sporadic cases is largely unclear. Idiopathic PD is believed to result from the complex interplay among multiple genes and environmental factors. Exposure to particular pesticides is associated with increased risk of PD^9^, while caffeine consumption can reduce PD risk^10^. Thus, large-scale epidemiological longitudinal data are needed to truly evaluate the risks of PD. Furthermore, genome-wide association studies (GWAS) have so far reported 41 PD-associated risk loci in various cohorts^11–13^, yet translation of the findings into biological understanding remains a major challenge and requires better and innovative tools to dissect the molecular mechanisms of PD.

The development of high-throughput molecular profiling techniques has advanced the research of complex diseases with detailed regulatory machineries hidden underneath. Gene network analysis has been extensively utilized as an unbiased approach to identify gene co-expression/co-regulation patterns in higher organisms for discovery of novel pathways and gene targets in various biological processes and complex human diseases^14–23^. For example, our previous study integrated large-scale genetic and gene expression data as well as clinical and pathological traits into multiscale network models of Alzheimer’s disease (AD)^16^, and several predicted key regulators of AD pathogenesis such as *TYROBP, DTL/CDT2*, and *GJA1* were subsequently validated in various model systems^24–27^. However, such network biology approaches have not been applied to PD research yet due to the lack of molecular profiling data from a large number of postmortem brain samples. As a result, little is known about the global as well as local structures of gene interactions and regulations in PD, thus hindering our understanding of idiopathic PD.

In this study, we develop multiscale gene network models of PD based on an ensemble of all the existing human brain gene expression data sets in PD followed by a comprehensive functional validation of a top key regulator. We identify a neuron-specific synaptic signaling gene module most associated with PD and pinpoint *STMN2* as one top key regulator of the module. Knockdown of *Stmn2* in mouse DA neurons leads to impaired synaptic vesicle (SV) endocytosis. Knockdown of *Stmn2* in the mouse SN further causes DA neuron loss, increased α-synuclein phosphorylation, and locomotor deficits. The network models not only shed a light on the global landscape of molecular interactions and regulations in PD but also reveal detailed circuits and potential key regulators of PD pathogenic pathways for further experimental investigation.

## Results

### An integrative multiscale network biology framework for PD

We employed an integrated network biology framework to systematically model a large transcriptomic data set in PD (Supplementary Fig. 1a). By searching the Gene Expression Omnibus (GEO, https://www.ncbi.nlm.nih.gov/geo/) database using Parkinson’s Disease and human substantia nigra as key words, we collected gene expression data from eight human PD studies^28–32^ (Supplementary Table 1). The curated gene expression data went through log2 transformation, quantile normalization, and correction for known covariates by a linear regression model. The differentially expressed genes (DEGs) between the case and control groups were identified by a meta-analysis followed by Benjamini–Hochberg (BH) correction. Gene ontology (GO) analysis was performed on the DEGs to identify dysregulated pathways in PD. The expression data from all the PD samples in the collection were merged into a global PD expression data set (*n* = 83) by Z-score transformation, and the same process was applied to the control samples (*n* = 70). The batch effect of these studies was removed by Z-score transformation as shown in Supplementary Fig. 1b. Subsequently, Multiscale Embedded Gene co-Expression Network Analysis (MEGENA)^20^ was performed on the global PD and control data sets separately. The co-expressed gene modules in the global PD network were then characterized by their enrichment for the PD DEGs and the cell-type specific markers^33,34^. In parallel, we constructed the module-based Bayesian regulatory networks (BN)^35,36^ to infer the regulatory relationships among the genes in each gene module. Key network hub genes in the top-ranked module were further prioritized based on differential expression and network neighborhood enrichment of the PD DEGs for experimental validation.

### PD DEGs are enriched for disease-associated pathways

We identified 946 DEGs between the PD and control groups in the meta-analysis of the eight curated data sets at 5% FDR and standard mean difference (SMD) >0.5 (Supplementary Data 1). To investigate whether the DEGs identified were associated with the clinical traits, we performed a separate meta-analysis to identify DEGs by excluding GSE49036^28^, which was the only data set in our collection with a complete clinical assessment and a sufficient number of samples for correlation analysis. This separate analysis yielded 420 DEGs (Supplementary Data 2), and 1,633 genes correlated with Braak score (Supplementary Data 3) and there was a significant overlap between the negative Braak-correlated genes (BCGs) and the downregulated DEGs (multiple-testing corrected Fisher’s Exact Test (cFET) *p* = 2.7E-54, 5.7-fold enrichment (FE)) as well as between the positive BCGs and the upregulated DEGs (cFET *p* = 9.5E-05, 4.3 FE) (Supplementary Fig. 2a, b), suggesting the robustness of meta-analysis to identify DEGs associated with disease progression. Therefore, we focused on the 946 DEGs identified in the meta-analysis of the eight curated data sets for the downstream analysis. Gene Ontology (GO) analysis (Supplementary Data 4) showed that the downregulated DEGs were enriched for synaptic function and metabolism-related GO terms, such as synaptic signaling, synaptic vesicle cycle, and organophosphate metabolic process, indicating that neuronal activity particularly neurotransmission was impaired in PD, while the upregulated DEGs were associated with spinal cord development and embryonic digit morphogenesis. These DEGs are largely consistent with the previously published meta-analyses in PD postmortem brains. Glaab and Schneider applied protein–protein interaction network (PPIN)-based enrichment analysis to identify synaptic transmission and dopamine metabolism as the top disrupted pathways in PD^37^. In Oerton and Bender’s analysis, 21 of the 32 top-ranked downregulated genes and 2 of the 11 top-ranked upregulated genes were present in our meta-analysis with consistent regulation (cFET *p* = 5.9E-19, 11.6 FE and cFET *p* = 0.02, 12.3 FE, respectively)^38^. Zheng et al. showed that electron transport chain, oxidative phosphorylation and metabolic pathways were most affected in PD^32^. Taken together, our meta-analysis provides a PD DEG signature that extends the published work for further analysis.

### PD MEGENA modules show cell-type-specific features

**Fig. 1:**
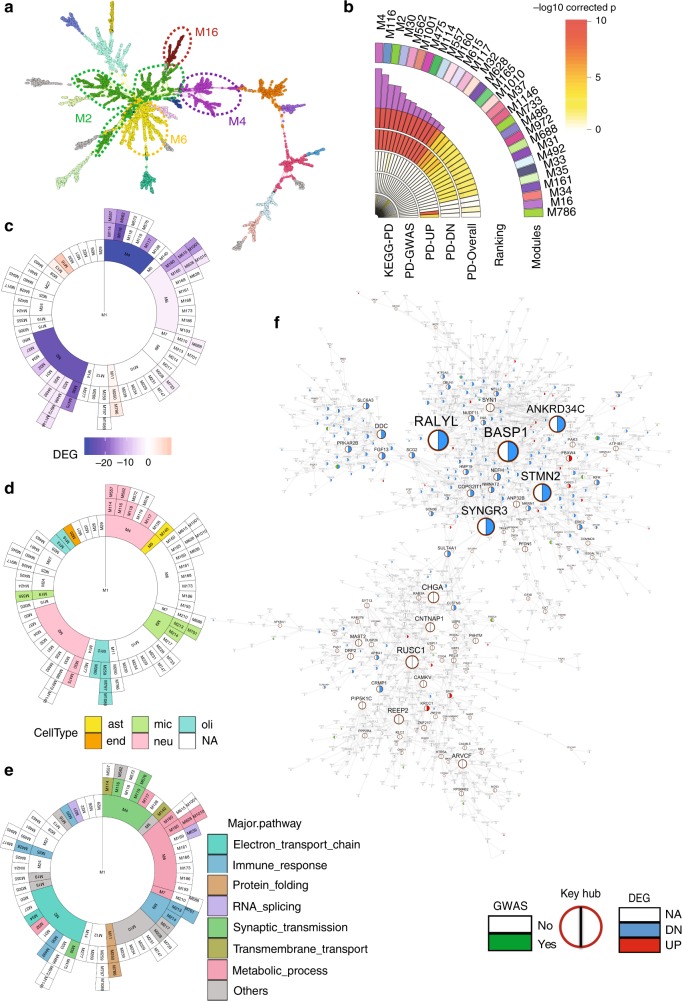
Gene co-expression modules associated with PD. **a** A global MEGENA network. The modules at one particular compactness scale are represented by different colors. Four parental modules (M4, M2, M6, and M16) most associated with PD are highlighted. **b** The top 30 MEGENA modules most enriched for the PD-related gene signatures including the PD DEGs, the PD GWAS genes, and the genes in the KEGG PD pathway. **c** Enrichment of the DEGs of PD in all the MEGENA modules. Color intensity is proportional to −log10(corrected FET *p*-value). **d** Enrichment of cell-type-specific gene signatures in all the MEGENA modules. Color intensity is proportional to −log10(corrected FET *p*-value). **e** Major pathways associated with the MEGENA modules. **f** The top-ranked module M4 is highly enriched for the downregulated DEGs in PD and the neuron-specific gene signatures. Key regulators are highlighted in burgundy circles. The pie chart of each node indicates whether it is a DEG or GWAS gene. The size of a node is proportional to the node connectivity in the MEGENA network

A total of 90 parent-child modules with module size >50 were identified from the global PD data set using MEGENA (Fig. 1a). These modules were then rank-ordered by their relevance to PD (Fig. 1b; Supplementary Table 2) which was calculated from the enrichment for the above identified DEGs (Fig. 1c), PD GWAS hits downloaded from GWAS catalogue (https://www.ebi.ac.uk/gwas/) and an independent set of PD genes curated from the Kyoto Encyclopedia of Genes and Genomes (KEGG hsa05012). Each module was annotated by the most significantly associated GO term (Fig. 1e for major pathways; for the complete list, see Supplementary Data 5).

As the central nervous system (CNS) is composed of multiple types of cells with distinct contributions to PD pathogenesis and progression, we examined the cell-type specificity of the modules using the gene signatures of six major brain cell types, including neurons, astrocytes, microglia, endothelial cells, oligodendrocyte precursor cells (OPC), and oligodendrocytes generated from the human brain^33,34^ (Fig. 1d; Supplementary Data 6). The results reflected the cell-type-specific functional modules of highly coordinated molecular dysregulations in PD. The module M4 was ranked as the top for its most significant enrichment of the overall PD DEGs (cFET p = 7.9E-26, 2.3 FE) and downregulated DEGs (cFET *p* = 2.7E-29, 2.7 FE) followed by its three child module branches M116-M562, M114-M557, and M117. M4 was associated with synaptic signaling (cFET *p* = 7.7E-05, 2.1 FE) and enriched for neuron-specific markers (cFET *p* = 6.2E-28, 3.9 FE). The other branches of the top-ranked modules included the M2 branch (M30-M475 and M32) and the M6 branch (M160-M615-M1001 and M165-M628-M1010). The M2-M30-M475 branch was also associated with synapse-related functions and enriched for neuron-specific markers (Supplementary Table 2; Supplementary Data 4). This is not surprising as multiple PD risk genes are associated with synaptic transmission, such as *LRRK2* and *SYNJ1*^6^. The M32 and M6-M160/M165 branches, although not showing any cell-type specificity, were significantly enriched for the downregulated PD DEGs and associated with electron transport chain and complex metabolic process, respectively, suggesting shared disease mechanisms across different cell types. On the other hand, M16 was most significantly enriched for the upregulated PD DEGs (Fig.1c) and the oligodendrocyte-specific markers (Fig. 1d; Supplementary Table 2), suggesting that oligodendrocytes play a significant role in the disease progression. The KEGG PD pathway (hsa05012) comprised 142 genes was enriched for the downregulated genes in PD (cFET *p* = 2.1E-07, 3.8 FE) and most enriched in the M2-M32 and M165-M628-M1010 branches (Supplementary Table 2).

### Key regulators of the top module are dysregulated in PD

To better understand the PD network, we investigated the underlying network topological structures of the top module, the neuron-specific M4 that was comprised of 947 genes and was enriched for the downregulated DEGs (Fig. 1f). As the links in the MEGENA network were undirected and represented gene co-expression or co-regulation, we further constructed a module-based BN using the same set of gene expression profiles from the PD patients to infer the potential regulatory relationships among genes, as we did in the previous study of AD^16^. Distinct from co-expression networks which are built upon correlations in the linear space, BNs are able to capture nonlinear relationships due to the data discretization and modeling on categorical distributions. Thus, BN can be used in complement to MEGENA for candidate discovery and prioritization. Network hubs identified in both the MEGENA network and the BN are likely to be more robust than those identified by a single network. We identified 33 and 45 key regulators of M4 by MEGENA and the key driver analysis (KDA) of the M4-based BN, respectively. The two sets of regulators shared 16 genes including 10 downregulated and 1 upregulated genes in PD in comparison with the normal control. We then rank-ordered these key regulators based on their enrichment of the PD DEGs in their two-layer network neighborhoods. The network neighborhood of a given gene refers to the genes that are *N*-layers (*N* = 1, 2, …) away from the gene in a given network. We identified *RALYL, BASP1, ANKRD34C, STMN2*, and *SYNGR3* as the top five key regulators of M4 by combining the rankings from both the MEGENA network and the M4-based BN (Supplementary Table 3). We hypothesize that perturbation of these key regulators of M4 could potentially influence the gene networks and ultimately introduce PD-like phenotypes. To further prioritize the key hub genes for experimental validation, we examined their differential expression in the human SN (Supplementary Fig. 4) and in different cell types in the adult mouse SN^39^. All of five top key regulators were downregulated in the human SN with PD, while only *Stmn2* and *Syngr3* had a preferable expression in the DA neurons over the other neuronal subtypes in the adult mouse SN (Supplementary Table 4). *Syngr3* homozygous knockout mice demonstrated a significant decrease in locomotor activity compared with their age- and sex-matched controls (Supplementary Fig. 5e, data downloaded from https://www.mousephenotype.org) and *Syngr3* mediated presynaptic dysfunction induced by Tau^40^, which was consistent with our prediction that *Syngr3* was involved in neurotransmitter transport (Supplementary Data 7). Therefore, we selected *STMN2* for further functional investigation.

### Network-specific functional annotation of PD GWAS genes

The links between the genes in the MEGENA network suggest high correlation and potential co-regulation in similar biological processes. Therefore, the network neighborhoods can be used to annotate biological functions of the known PD risk genes. The PD MEGENA network includes 125 known PD GWAS genes curated in the GWAS catalog (https://www.ebi.ac.uk/gwas/). For each GWAS gene, we assigned the top pathway enriched in its two-layer network neighborhood as the putative annotation (Supplementary Data 8). For example, *AAK1* was predicted to be associated with synaptic signaling (cFET = 0.023, 4.4 FE), and indeed it was reported to regulate clathrin-mediated endocytosis^41^. Such contextual annotation of the PD GWAS genes can be applied to any gene in the network to understand their disease-specific function. For example, *STMN2*, one top hub in M4 and a known regulator of microtubule dynamics^42–44^, was predicted to be highly associated with synaptic signaling according to our network based annotation (cFET *p* = 1.9E-04, 4.4 FE; Supplementary Data 7).

**Fig. 2:**
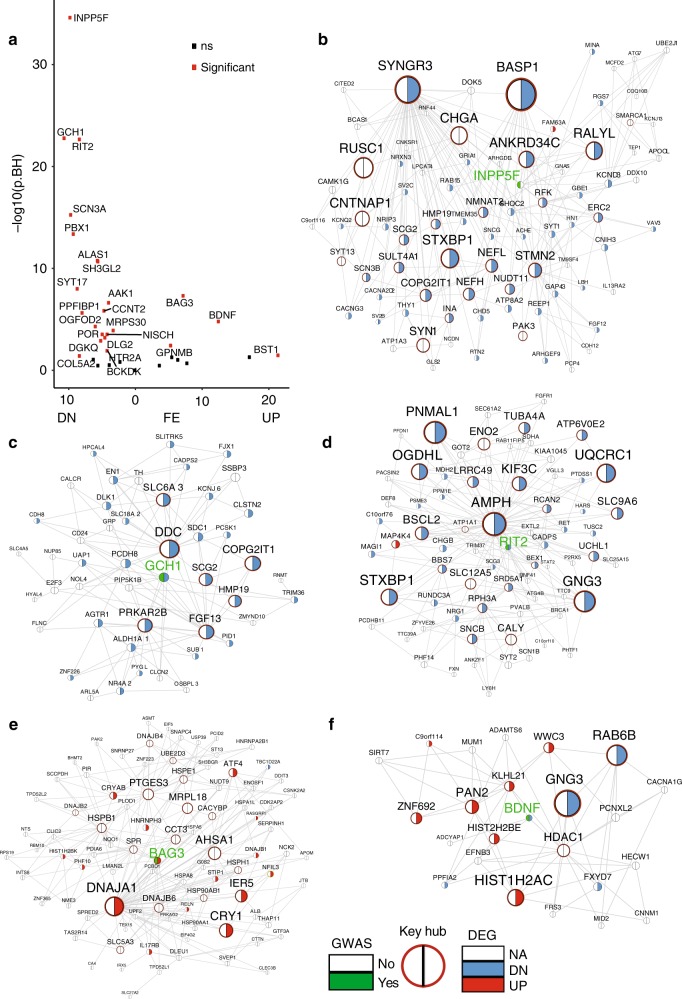
The subnetworks of the top-ranked PD GWAS genes. **a** Enrichment of the PD DEGs in the network neighborhoods of a subset of the PD GWAS genes. **b**–**e** The network neighborhoods of *INPP5F, GCH1, RIT2, BAG3,* and *BDNF*, respectively. The pie chart of each node indicates whether it is a DEG or GWAS gene. The size of a node is proportional to the node connectivity in the MEGENA network

In addition, we examined how the network neighborhood of each PD GWAS gene was enriched for the PD DEG signature (Fig. 2a; Supplementary Data 9). Strong enrichment indicates more functional involvement in PD pathogenesis beyond the implicated genetic association with the disease. The top GWAS genes whose network neighborhoods were most significantly enriched for the genes downregulated in PD versus control were *INPP5F, GCH1*, and *RIT2* (Fig. 2b–d) and the latter two were downregulated in PD (Supplementary Data 1). The top two GWAS genes, whose network neighborhoods were highly enriched for the genes upregulated in PD, were *BAG3* (Fig. 2e), which was upregulated in PD, and *BDNF* (Fig. 2f), which was downregulated in PD (Supplementary Data 1). *STMN2* fell into the neighborhood of *INPP5F* (Fig. 2b). Although *STMN2* is not a known genetic risk factor for PD, our results indicate that *STMN2* is functionally linked to some PD risk genes and potentially plays a critical role in mediating molecular alterations in PD.

### *Stmn2* knockdown causes presynaptic dysfunction in DA neurons

**Fig. 3:**
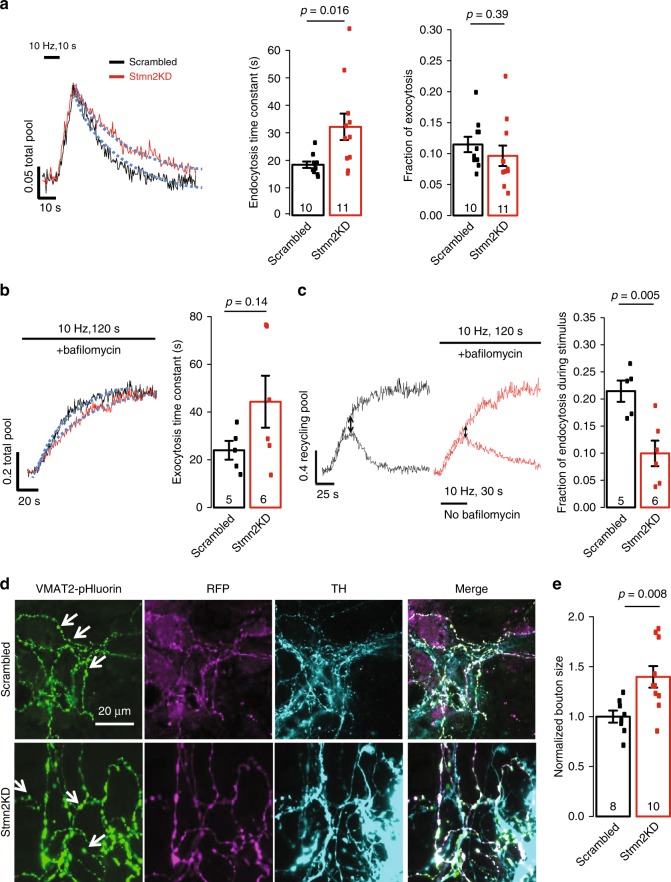
pHluorin assay in cultured DA neurons with *Stmn2* knockdown. **a** SV endocytosis and exocytic fraction in midbrain DA neurons with or without *Stmn2* knockdown. Two-sided Student’s *t* test for two-group comparison. *t* = −2.6572, df = 19, *p*-value = 0.01556 for endocytosis time constant, which is the τ of the fitted one-phase exponential decay indicated by the blue dash line; *t* = 0.87198, df = 19, *p* = 0.3941 for exocytic fraction, which is the normalized peak height. **b** SV exocytosis in midbrain DA neurons with or without *Stmn2* knockdown. The exocytosis time constant is the τ of the fitted one-phase exponential decay indicated by the blue dash line. This is a subset of neurons recorded in (**a**). Two-sided Student’s *t* test for two group comparison. *t* = −1.6213, df = 9, *p*-value = 0.1394. **c** SV endocytosis during stimulation in midbrain DA neurons with or without *Stmn2* knockdown as indicated by the arrows in **c**. This is a subset of neurons recorded in (**a**) and the same neurons in (**b**). Two-sided Student’s *t* test for two group comparison. *t* = 3.6414, df = 9, *p*-value = 0.005389. *N* indicated in each experiment. **d** Immunostaining of VMAT2-pHluorin, RFP tag of Stmn2-shRNA plasmid, and TH of recorded DA neurons treated with scrambled or *Stmn2*-shRNA. Arrows pointed to representative boutons. **e** Quantification of bouton sizes in scrambled and *Stmn2*-shRNA-treated TH + neurons. Two-sided Student’s *t* test for two group comparison. *t* = 3.2224, df = 16, *p*-value = 0.008433. All data are present as mean ± SEM. Source data are provided as a Source Data file

*Stmn2* is known to regulate microtubule dynamics, axon formation and neurite outgrowth during development^42–44^ and regeneration after injury^45^, while we predict that *Stmn2* may regulate presynaptic activity in PD (Supplementary Data 7). To test the hypothesis, we employed a live cell imaging assay utilizing pHluorin (described in the Methods section) in cultured midbrain DA neurons where *Stmn2* was highly expressed^39^ (Supplementary Fig. 5a, b). pHluorin is a variant of green fluorescence protein (GFP) sensitive to pH. When targeted to the acidic lumen of synaptic vesicles, pHluorin is quenched but fluoresces upon exocytosis when exposed to the extracellular buffer (pH 7.4). Conjugating pHluorin to vesicular transporters, such as vesicular glutamate transporter 1 (vGLUT1) or vesicular monoamine transporter-2 (vMAT2) was used to examine synaptic vesicle kinetics in a quantitative manner^46–48^. DA neurons expressing CMV-vMAT2-pHluorin were recorded at 9–11 days after *Stmn2*-shRNA transfection. *Stmn2*-shRNA-treated DA neurons demonstrated impaired SV endocytosis after and during stimulation compared with scrambled shRNA-treated DA neurons (Fig. 3a, c), while the exocytosis was not significantly affected (Fig. 3a, b). We also observed enlarged boutons in *Stmn2* knockdown DA neurons where the pHluorin assay was recorded (Fig. 3d, e), while no obvious degeneration of the soma or cell death was seen at the time of recording. Interestingly, bouton size was not significantly affected in the cultured TH-negative neurons (Supplementary Fig. 5d, e). Our data thus corroborate the network based prediction and points to a key role of *STMN2* in regulating presynaptic activity.

### Knockdown of *Stmn2* perturbs *STMN2*-centered subnetworks

**Fig. 4:**
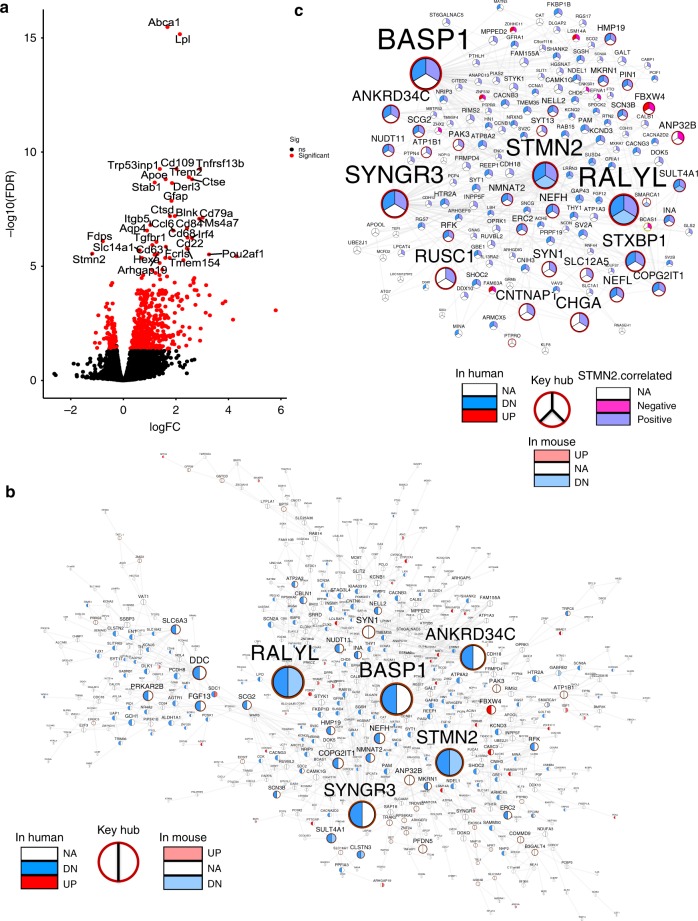
Validation of the PD MEGENA network by *Stmn2* perturbation. **a** Volcano plot shows genes that are significantly differentially expressed in the midbrain between the mice received scrambled AAV and *Stmn2*-shRNA AAV. **b** The genes in M4 are significantly enriched for the downregulated DEGs identified in the midbrain of the *Stmn2* knockdown mice. Nodes with burgundy labels are key hubs of M4. The left-side half circle of each node indicates the DEGs in human between the PD and control groups while the right-side represents the DEGs in mice by *Stmn2* knockdown. **c** The two-layer network neighborhood of *STMN2* is significantly enriched for the DEG downregulated by *Stmn2* knockdown. Nodes with burgundy labels are key hubs in the MEGENA network. The left upper part of the circle represents the DEG in human between the PD and control groups while the right upper part of the circle represents the correlation with *STMN2* in PD patients. The bottom part of the circle indicates the DEGs in mice by *Stmn2* knockdown

We then sought to validate in vivo whether the perturbation of *STMN2*, one of the top key regulators in the top-ranked module M4 would preferentially induce changes of the gene expression in *STMN2*’s network neighborhoods as well as *STMN2*-correlated genes in PD. To mimic the decreased *STMN2* expression in PD brains (Supplementary Fig. 4), we performed genetic knockdown in mouse brains with a red-fluorescent-protein (RFP)-tagged Adeno-associated virus (AAV2/1) containing the validated *Stmn2* shRNA sequence. After confirmation of the knockdown efficiency in N2A cells and wild-type mouse brains (Supplementary Fig. 6), we performed a unilateral injection of the virus into the right SN of 2-month-old male C57BL/6J mice. The injected mice were sacrificed at 1 month post injection, and the infected SN was isolated for RNA sequencing. DEG analysis identified 527 upregulated and 115 downregulated genes caused by *Stmn2* knockdown (fold change > 1.2 and FDR < 0.05; Fig. 4a; Supplementary Data 10). *Stmn2* itself was most downregulated, indicating the knockdown efficiency. The genes downregulated by *Stmn2* knockdown were associated with sterol/lipid biosynthesis and metabolism (cFET *p* = 1.4E-10, 59.2 FE), while those upregulated by *Stmn2* knockdown were associated with immune/inflammatory response (cFET *p* = 3.9E-44, 3.6 FE). As *Stmn2* is not known as a transcription factor or cofactor, the upregulation of these immune-related genes is likely to be secondary. Specifically, knockdown of *Stmn2* upregulated nine PD GWAS genes (*GPNMB*, *SREBF1, STAB1, LHFPL2, PRRG4, CTSB, FNDC3B, PPFIBP1*, and *COL5A2)*, and downregulated one PD GWAS gene (*SYT4*) at the mRNA level. While *STAB1, LHFPL2, PRRG4, FNDC3B, PPFIBP1*, and *COL5A2* were not reported to be functionally related to PD, we predicted that they were associated with immune response, oxidoreductase activity, and EGF pathway annotated based on their network neighborhoods. Indeed, *GPNMB* was elevated in the SN of human PD brains and played an anti-inflammatory role in a CD44-dependent manner^49^. *SREBF1*, a key regulator of the lipogenesis pathway, was found to stabilize *PINK1* during mitophagy initiation^50,51^. *CTSB*, known to encode cysteine cathespsin B, was essential in lysosomal degradation of α-synuclein^52^. Upregulation of these PD GWAS genes suggested a compensatory mechanism in response to *Stmn2* deficiency, while *SYT4*-mediated somatodendritic release of DA was a key mechanism for the autoregulatory control of DA release in the brain^53^.

We then examined how *Stmn2* knockdown impacted *STMN2*-correlated genes identified in the PD patients (Supplementary Data 11). After mapping mouse genes to the human homologues, we found a significant overlap between the genes positively correlated with *STMN2* in PD patients and those downregulated in *Stmn2* knockdown mice (cFET *p* = 1.4E-27, 5.7 FE) and between the genes negatively correlated with *STMN2* in the PD patients and the genes upregulated in *Stmn2* knockdown mice (cFET *p* = 0.04, 1.6 FE). The DEGs downregulated in *Stmn2* knockdown mice were significantly enriched in the module M4 (FET *p* = 0.015, 2.0 FE, Fig. 4b) as well as in the two-layer MEGENA network neighborhood of *STMN2* (FET *p* = 4.3E-06, 8.6 FE; Fig. 4c), while the upregulated DEGs in *Stmn2* knockdown mice were not significantly enriched in either M4 or *STMN2*’s MEGENA network neighborhood. As the majority of the DEGs in *Stmn2* knockdown mice were upregulated and involved in immune response, which was absent in the meta-analysis of the data sets from the postmortem PD brains, we posited that this *Stmn2* knockdown mouse model might partially recapitulate the PD pathogenic processes in the human brain.

### Knockdown of *Stmn2* impairs locomotor functions in mice

**Fig. 5:**
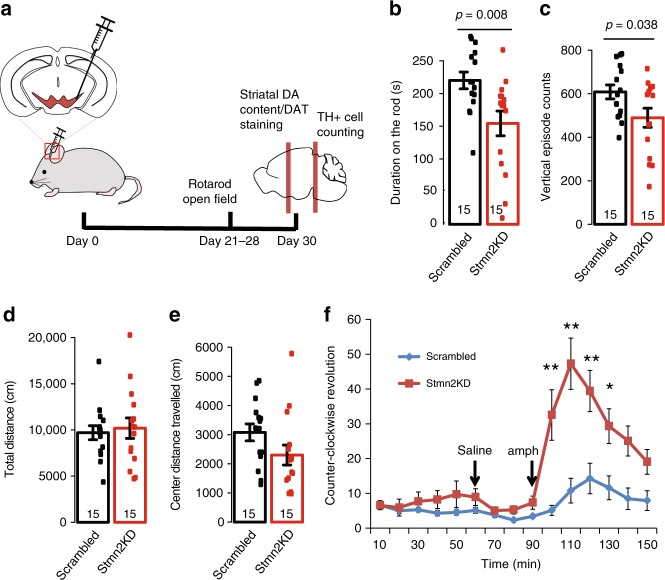
Locomotor behavioral analysis in mice with AAV-mediated *Stmn2* knockdown. **a** The schematic of the experimental design. AAV carrying scrambled shRNA or *Stmn2*-targeting shRNA was injected in the right SN. Behavioral tests were performed from day 22 to day 27 post injection. **b** The injected mice were examined on the rotarod. The average of three trials was used to represent the performance of each individual mouse. *N* = 15 in each group. Student’s *t* test, two-sided, *t* = 2.873, df = 28, *p*-value = 0.008. **c**–**e** The injected mice were examined in open field. The vertical episode count (**c** *t* = 2.1851, df = 28, *p*-value = 0.038), total distance (**d** *t* = −0.36436, df = 28, *p*-value = 0.3592), and center distance traveled (**e** *t* = 1.7304, df = 28, *p*-value = 0.094) were compared between the control and *Stmn2* knockdown mice. *N* = 15 in each group, two-sided Student’s *t* test for two group comparison. **f** The injected mice were also examined in open field at baseline, under saline injection, and under amphetamine injection. The counter-clockwise revolution was compared between the control group and *Stmn2* knockdown group. *N* = 15 in each group, two-way ANOVA, df = 1, F = 75.951, *p* = 2E-16 between control and *Stmn2* knockdown; df = 14, F = 13.669, *p* = 2e-16 between time points and df = 14, F = 6.244, *p* = 2.27E-11 for interacting terms; post hoc comparison was performed using Tukey HSD test *<0.05, **<0.01. All data are present as mean ± SEM. Source data are provided as a Source Data file

We next examined whether knockdown of *Stmn2* could induce PD-like behavioral and pathological changes. We performed a unilateral injection of AAV2/1 carrying either the *Stmn2*-targeting shRNA or a scrambled sequence into the SN of 2-month-old male mice. We assessed the locomotor activity by performing Rota-rod and open field tests in the fourth week after injection (Fig. 5a). The mice with *Stmn2*-shRNA injection spent significantly less time on the rod than the mice with scrambled shRNA injection (Student’s *t* test *p* = 0.008, Fig. 5b). In open field, the *Stmn2*-shRNA-treated mice showed fewer vertical episode counts than scrambled shRNA-treated ones (Student’s *t* test *p* = 0.038, Fig. 5c), while there was no difference in the total distance between the two groups (Fig. 5d) and a trend toward shorter center distance traveled (Fig. 5e) in the *Stmn2* knockdown mice. As the injection was given in mice unilaterally, administration of amphetamine could induce rotational asymmetry in mice, which was commonly used for testing DA-dependent progression in locomotor function lesion^54^. The amphetamine treatment indeed induced a significant increase in contralateral rotation in the *Stmn2*-knockdown mice (Fig. 5f). Taken together, our data suggested that *Stmn2* knockdown caused DA related locomotor dysfunction.

### *Stmn2* knockdown reduces striatal DA content and DA terminals

**Fig. 6:**
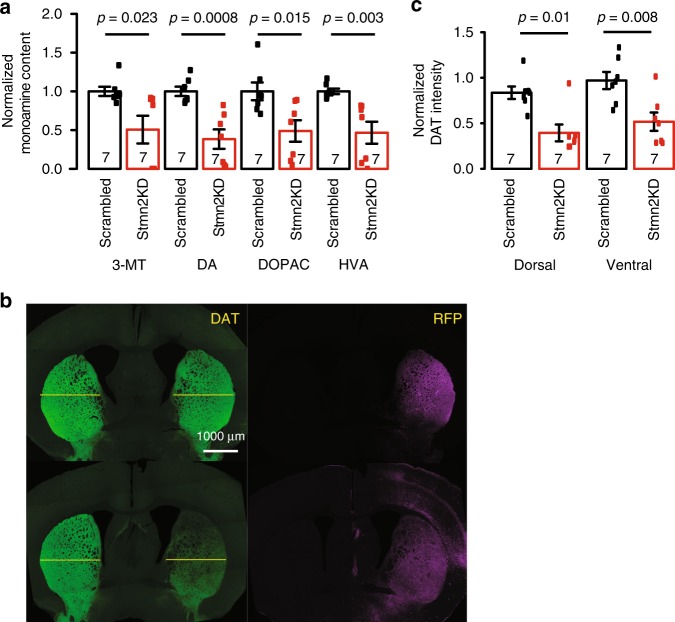
Striatal alterations in mice with AAV-shRNA-mediated *Stmn2* knockdown. **a** The content of dopamine (DA) and its major metabolites (3-MT, DOPAC, and HVA) were measured in the contralateral and ipsilateral striatal tissues isolated from the mice that received scrambled AAV and *Stmn2*-shRNA AAV injections, *N* = 7 in each group. The biogenic monoamine levels of the ipsilateral striatum were normalized to those of the contralateral side in each individual mouse, and the comparison between mice with scrambled and *Stmn2*-shRNA AAV injection was analyzed by two-sided Student’s *t* test. DA: *t* = 4.4367, df = 12, *p*-value = 0.0008; DOPAC: *t* = 2.8365, df = 12, *p*-value = 0.01499; 3-MT: *t* = 2.614, df = 12, *p*-value = 0.02263; HVA: *t* = 3.6415, df = 12, *p*-value = 0.003379. **b** Anti-DAT antibody was used to detect DAT + terminals in the striatum in mice that received scrambled AAV and *Stmn2*-shRNA AAV injections. RFP serves as an indicator of transfected terminals. The yellow line separated the dorsal and ventral striatum. **c** The quantitative analysis of DAT + immunofluorescent intensity in the two groups of mice. The DAT fluorescence intensity of ventral striatum without injection was used as the baseline to normalize the DAT intensity of the rest regions. *N* = 7 in each group. Two-way ANOVA, *F* = 25.689, df = 25, *p* = 3.12E-05. All data are present as mean ± SEM. Source data are provided as a Source Data file

We further examined the striatal DA content by performing high-performance liquid chromatography (HPLC). We found a 50% reduction of DA as well as the three major metabolites (DOPAC, 3-MT, and HVA) in the ipsilateral (right) striatum compared with the contralateral (left) side in the mice with *Stmn2* shRNA injection (Fig. 6a). We also examined the density of dopamine transporter positive (DAT+) terminals in the dorsal and ventral striatum based on DAT fluorescence intensity. We noticed a reduction of DAT+ terminal density in both the dorsal and ventral striatum of the ipsilateral side in the mice with *Stmn2* shRNA injection compared with that in the mice with scrambled shRNA injection (Fig. 6b, c), suggesting the knockdown of *Stmn2* led to the terminal degeneration of DA neurons in both SN and the ventral tegmental area (VTA). By tracing the AAV RFP tag, we confirmed the viral spreading into the SN-adjacent brain regions, including VTA (Supplementary Fig. 6h).

### *Stmn2* knockdown leads to DA neuron loss

**Fig. 7:**
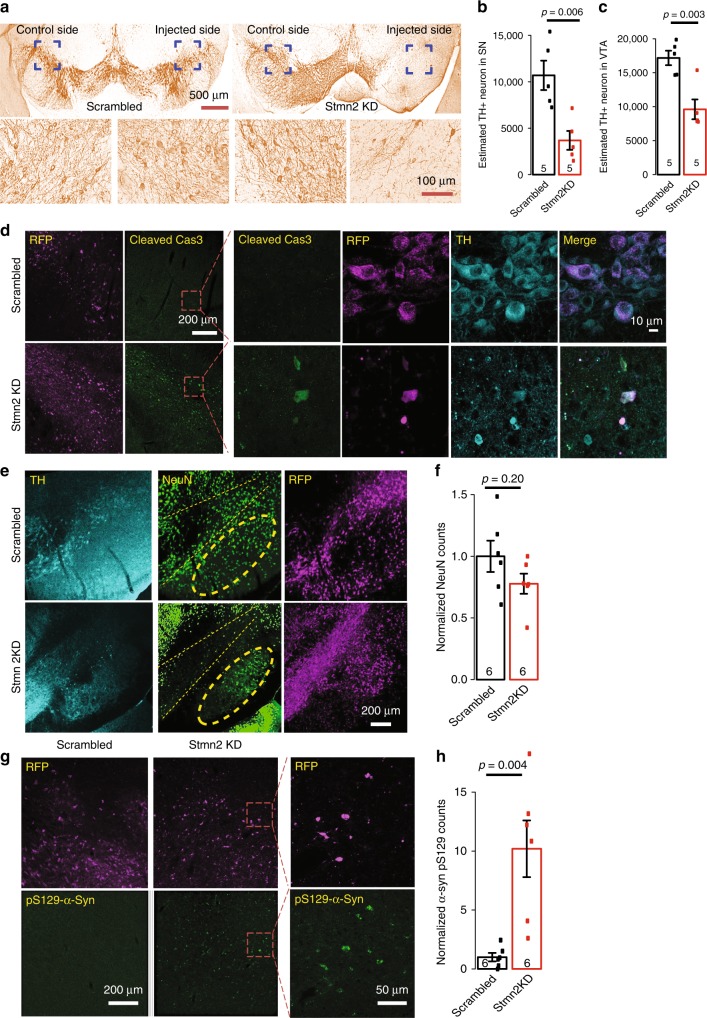
Pathological characterization in the SN of mice with *Stmn2* knockdown. **a** Immunohistochemistry staining with anti-TH antibody in the SN from mice injected with AAV carrying scrambled or *Stmn2* shRNAs. **b** Stereological counting of TH+ neurons in SN of mice injected with AAV carrying scrambled or *Stmn2* shRNAs and the result was analyzed by two-sided Student’s *t* test. *N* = 5 in each group. *t* = 3.7256, df = 8, *p*-value = 0.005824. **c** Stereological counting of TH+ neurons in VTA of mice injected with AAV carrying scrambled or *Stmn2* shRNAs and the result was analyzed by two-sided Student’s *t* test. *N* = 5 in each group. *t* = 4.1854, df = 8, *p*-value = 0.003057. **d** Immunofluorescent staining with anti-cleaved caspase-3 and TH antibody in the SN of mice injected with AAV carrying scrambled or *Stmn2* shRNA. Red dash squares were zoomed in with higher magnification. Representative images from *N* = 6 in each group. **e**, **f** Immunofluorescent staining with anti-NeuN and anti-TH and quantification of TH−/NeuN+ cells in the SNr of mice injected with AAV carrying scrambled or *Stmn2* shRNA. SNr highlighted by yellow dash circle and SNc highlighted between two yellow dash lines. Two-sided Student’s *t* test, *N* = 6 in each group, *t* = 1.362, df = 10, *p* = 0.2031. **g**, **h** Immunofluorescent staining with anti-pS129 of α-synuclein (pS129-α-syn) antibody and quantification of pS129-α-syn puncta in the SN of mice injected with AAV carrying scrambled or *Stmn2* shRNA. Red dash squares were zoomed in with higher magnification. Representative images from *N* = 6 in each group. Two-sided Student’s *t* test *p* = 0.004, *t* = −3.7825, df = 10, *p* = 0.004. All data are present as mean ± SEM. Source data are provided as a Source Data file

We then asked if impaired locomotor activity and reduced striatal DA content were associated with the loss of DA neurons by performing stereological counting of TH+ neurons of the substantia nigra compacta (SNc) and VTA in the injected mice. We found a striking (~70%) reduction of TH+ neurons in the SNc of the mice with *Stmn2*-shRNA injection compared with the mice with scrambled shRNA injection (Fig. 7a, b). A 40% reduction of TH+ neurons in the VTA was also observed (Fig. 7c). Increased cleaved caspase-3 immunostaining was found in the *Stmn2*-shRNA injected midbrain (Fig. 7d), confirming that the reduction of TH+ cell number was likely due to apoptotic cell death. We also examined the TH-/NeuN+ neurons in the substantia nigra reticulata (SNr) and found no significant cell loss in this region (Fig. 7e, f), suggesting that TH+ neurons were particularly vulnerable when *Stmn2* was depleted.

### *Stmn2* knockdown increases α-synuclein Ser129 phosphorylation

To investigate further the link between *Stmn2* and PD, which was pathologically associated with aggregated or modified α-synuclein, we examined the Ser129 phosphorylation of α-synuclein, a hallmark of synucleinopathy lesions in human^55^ via immunofluorescence. We observed puncta of phosphorylated α-synuclein proteins detected with anti-pSer129 α-synuclein antibody only in the *Stmn2* shRNA mice, but not in the scrambled shRNA mice (Fig. 7g, h), indicating that reduced *Stmn2* expression caused an increase of Ser129 phosphorylation of α-synuclein, a critical toxic modification of α-synuclein related to idiopathic PD.

## Discussion

Our multiscale network analysis of the gene expression data from a large number of postmortem PD brains has revealed network structures and key regulators functionally connected to known PD risk genes. Functional validation of one top-ranked key regulator *STMN2* demonstrates its critical role in controlling DA neuron function/viability and regulating α-synuclein modification, which are highly relevant to PD pathogenesis.

The emergence of systems/network biology has revolutionized the discovery of novel mechanisms and promising drug targets for complex diseases, such as cancer, diabetes, and AD^16,56,57^. The lack of large-scale molecular profiling data from PD postmortem brains has impeded the application of such systems biology approaches to PD. Although many gene expression studies of PD postmortem brains have been reported, each individual study involves a small number of samples and thus each alone is insufficient to derive a holistic picture or detailed signaling circuits of PD-related pathways. Here, we sought to address the above unmet challenge in PD research by assembling the gene expression profiles of the postmortem human brains from eight published PD studies into one gene expression data set, which enabled co-expression and causal network inference to systematically uncover intrinsic network structures and key regulators of PD. The comparison between the MEGENA modules and those from the traditional Weighted Gene Co-expression Network Analysis (WGCNA) has shown that MEGENA could provide consistent yet complementary information at a higher resolution in the PD context (See Supplementary Note 1 and Supplementary Data 12). Different from the simple clustering analysis offered by WGCNA, MEGENA can identify more coherent and functionally relevant modules with high-resolution topological network structures and clearly defined key regulators for downstream analyses. By investigating the neighborhood of a gene within the gene co-expression and causal networks, we were able to annotate and predict biological functions for each gene in a disease-specific manner, thus complementing the current GWAS studies with functional contexts.

The network modules that are most associated with PD contain genes involved in a number of diverse pathways, including synaptic transmission, oxidative phosphorylation, mitochondrion, myelination, and response to unfolded protein. *STMN2* is one key regulator of the top PD-associated module. *STMN2* has an overall reduced expression in the PD brains, and is predicted to play a role in synaptic transmission. *STMN2*, also known as superior cervical ganglion 10 (SCG10), is a ~20 kDa phosphoprotein of the stathmin family. Overexpression of Tau downregulated *Stmn2* in PC12 cell line^58^; administration of 1-methyl-4-phenyl-1,2,3,6-tetrahydropyridine (MPTP) also caused downregulation of *Stmn2* level and the rescue of MPTP-induced PD-like phenotype restored *Stmn2* expression in mice^59^, implicating a potential association between *Stmn2* and PD. Despite its known functions in regulating microtubule dynamics during development and after injury, knockdown of *Stmn2* led to impaired SV endocytosis in cultured DA neurons. Furthermore, the direct perturbation of *Stmn2* in the mouse SN recapitulated the gene expression pattern and the network structure identified from the SN of the PD subjects. Moreover, perturbation of *Stmn2* expression in mouse midbrain led to PD-like pathologies and behavioral abnormality, though the underlying mechanisms remain to be characterized.

Unlike monogenic form of PD, idiopathic PD is polygenic and has a complex etiology with interaction with environmental factors. In addition to *STMN2*, which we provide proof-of-principle validation of PD relevance in our study, we also predict many other putative regulators that could contribute to sporadic PD pathogenesis. For example, *BASP1* belongs to the family of growth-associated proteins, which also includes *GAP43/BASP2*. *Basp1*^−/−^ mice exhibited high postnatal mortality but can be rescued by overexpression of *Gap43*, suggesting a functional similarity between the two genes^60^. Interestingly, *Stmn2* knockdown in the mouse SN led to a decrease in *Gap43* but not *Basp1* at the mRNA level, implicating a functional difference between *Basp1* and *Gap43*. *RALYL*, another top-ranked regulator, is reported to be an interacting protein of LRRK2 in a yeast 2 hybrid analysis^61^. *RALYL* was downregulated by *Stmn2* knockdown in vivo. Reduced expression of *RALYL* has been implicated in worse prognosis in clear-cell renal cell carcinoma^62^ but its function in the human brain remains unclear. Thus, our study begins to reveal a global landscape of gene interaction and regulatory circuits underlying PD pathogenesis.

Distinct from the causal genetic variants in PD, many of the hub genes identified in our PD network have not been reported to be genetically linked to the disease, including our top hub gene of interest, *STMN2*. However, these key hub genes are predicted and/or confirmed to govern the expressions of many other genes, including disease-associated GWAS genes in the disease-specific networks and therefore are considered as drivers that can modify disease onset and progression. Indeed, we previously identified *TYROBP* as a key driver in AD pathogenesis^16^, and yet no SNPs are known to be associated with sporadic AD in the *TYROBP* locus. *TYROBP* has been identified as a binding partner for *TREM2*, *CD33*, and *CR3* with increased expression in AD patients^16^. Knockout of *Tyrobp* was neuroprotective in a mouse model of early Alzheimer’s pathology^63^. Therefore, they may serve as novel drug targets in addition to the disease-causing genetic variants. Our validation experiments provide strong evidence supporting a role for the key regulator *Stmn2* in modulating PD-related pathogenesis. Our future experiments will include assessment of other predicted key regulators of PD and discovery of novel compounds that can restore the expression or activity of key regulators of PD.

In summary, our integrative network biology approach has systematically uncovered a number of gene subnetworks and key regulators of PD. Our study of postmortem PD brains, however, offers only a snapshot at the late stage of the disease where the majority of DA neurons degenerated. This limitation poses hurdles to directly reconstruct molecular processes of the disease progression. The incomplete clinical assessment and limited number of samples in the published cohorts also prevent us from conducting stringent association study of the molecular data with clinical and pathophysiological traits. Our approach is expected to be improved with comprehensive longitudinal studies, increased sample size, and better diagnosis of PD. Nonetheless, our study provides a proof of principle of an integrated systems approach to a complex disease. It is imperative that future investigation should develop a large and coherent multi-Omics cohort from PD brains with high quality genetic, epigenetic, transcriptomic, and proteomic data, which will ultimately enable the discovery of more robust regulators for idiopathic PD.

## Methods

### Data collection and analysis

Publicly available data sets comparing the expressional difference between Parkinson’s disease patients and controls were downloaded from GEO using two key words, including Parkinson’s disease and human substantia nigra. Only studies with sample size >= 5 in each group were included. Altogether we obtained eight data sets that focused specifically on SN, as listed in Supplementary Table 1. The downloaded gene expression profile of each data set was already preprocessed using either RMA or MAS. Out of the eight data sets we collected, only one (GSE8397) has two regions (lateral and medial SN) assayed for the same subject and both of them were from SN. We used the lateral SN to represent the gene expression within the SN as the original publication claimed that lateral SN is more vulnerable in PD^29^. Thus we ensured that each sample in our analysis was from different individuals. The data were log2 transformed, quantile normalized, and corrected for covariates, such as gender and age etc. using a linear regression model lm(expression~as.factor(gender) + as.numeric(age) + as.numeric(RIN)) in R/3.4.3, if the information was available. As the samples were profiled from different platforms with unique probe sets, we investigated the expression on the gene level. For genes with multiple probes measured, we chose the probe with the largest variance to represent the gene expression.

### DEG analysis and Braak correlation analysis

Metafor 2.0.0^64^ was used to perform meta-analysis to identify differential gene expression across the eight data sets in our collection. For each gene profiled in all the eight data sets, we calculated the standardized mean difference (SMD) between the PD and control groups across all the data sets and then tested for heterogeneity using metafor package in R. If the data are significantly heterogeneous (*p* < 0.05), a random effect model was fitted; if not, a fixed effect model was fitted. BH correction was used to control multiple-testing errors. Genes with FDR < 0.05 and mean SMD > 0.5 were considered as differentially expressed genes (DEGs). We chose the cutoff based on the guideline by Cohen et al.^65^ that SMD = 0.5 represented a medium effect size. In one specific data set GSE49036, the gene expression data came together with complete Braak stage assessment. Therefore, we performed Spearman correlation analysis between the gene expression and the Braak score to identify BCGs (BH-corrected *p*-value < 0.05). Note that in this data set, samples from patients with incidental Lewy Body Disease (iLBD) were excluded for the DEG analysis, but included for Braak correlation analysis.

### Network construction and key regulator identification

Each QCed data set was split into disease and control groups. Z-score transformation was applied for each gene across all the samples (both disease (PD) and control samples) within each data set to control possible biases from different platforms and processing methods. We then merged the transformed data into a global disease data set (*n* = 83) or a control (*n* = 70) data set. Module detection and key hub identification were done in the disease-specific expression profile using MEGENA^20^. Modules were ranked based on enrichment with PD DEGs.

BN was constructed from genome-wide gene expression profile through a Monte Carlo Markov Chain (MCMC) simulation-based procedure by using software RIMBANET^35,36^. We made use of known transcription factor (TF)-target relationships derived from brain tissue-related cell types from the ENCODE project^66,67^. The TFs were allowed to be parental nodes of their targets, but the targets were not allowed to be parental nodes of the TFs. Such relationships were used as structural priors to assist with the topological computation. We followed a network averaging strategy with which 1000 networks were generated from the MCMC procedure starting with different random structure, and links that shared by more than 30% of the networks were used to define a final consensus network structure. An iterative de-loop procedure was executed to ensure that the consensus network was a directed acyclic graph, removing the most-weakly supported link of all links involved in any loop. Following Zhang et al.^16^, we performed KDA on the consensus Bayesian network to identify key hub genes which regulated a large number of downstream nodes.

To identify WGCNA modules, we first determined the soft power beta to be 4.5 as it gave the optimal fit for a scale free topology (*R*^2^ = 0.98, slope = −2.67). We then determined the module membership by the dynamic cutting tree algorithm implemented in the R package WGCNA. This process yielded 24 well-separated modules with a minimal size of 20.

The hub genes were first evaluated for the enrichment of the PD DEGs in their network neighborhoods in the MEGENA and BN networks separately, and the two enrichment scores for each hub were then combined into a final ranking score *G*_*j*_ = ∏_*i*_*g*_*ji*_, where *g*_*ji*_ is the discriminant value of a key hub gene *j* in the network *i*, defined as (*max*_*j*_(*r*_*ji*_) + 1 − *r*_*ji*_)/∑_j_*r*_*ji*_ and *r*_*ji*_ is the ranking of the enrichment score for the key hub gene *j* in the network *i*^16^. A smaller *r*_*ji*_ indicates a higher rank of a gene with a larger enrichment score. A gene with a larger final score *G*_*j*_ is considered as a more important key hub gene in both networks.

### Gene set enrichment analysis

Modules and network neighborhood genes of key hubs were intersected with GO terms, signaling pathways, and cell-type specific expression gene signatures. The significance level of the overlap between two gene sets was determined by Fisher’s exact test. For all the module-related enrichment analyses, the genes in all the modules were used as the background. For the network neighborhood enrichment analysis, all the genes in a given network (i.e., the MEGENA or Bayesian network in PD) were taken as the background. For GO analysis, the genes shared by a query gene set (e.g., the genes in all the modules, all the genes in the MEGENA network and so on) and the set of all the genes present in the GO database were used as the background. The size of the background used for the abovementioned enrichment analyses can be found in the associated Supplementary Tables, i.e., the column background size. The *p-*values were computed based on the hypergeometric distribution followed by BH correction for multiple testing.

### Cell cultures and shRNA transfection

Mouse N2A neuroblastoma cells (ATCC CCL-131) were cultured in DMEM supplemented with 10% fetal bovine serum (FBS) at 37 °C with 5% CO_2_. Approximately 80,000 cells were seeded into 12-well plates 24 h before lipofectamine 3000 (Life Technology, CA, USA) transfection. Seventy-two hours after transfection, cells were collected in RIPA buffer for western blot.

The Institutional Animal Care and Use Committee (IACUC) of Icahn School of Medicine at Mount Sinai approved all the animal-related experiments. We have complied with all relevant ethical regulations for animal testing and research. Mouse midbrain neurons were cultured from P0-P1 wild-type C57BL/6J mice (Jackson Laboratory, ME, USA). Typically, four P0-P1 mouse brains were required for a midbrain culture. Only the ventral midbrain containing SN and VTA was collected to enrich the TH-positive population (~30% of the total neurons per culture dish based on our estimate). The brain tissues were then incubated with papain (Worthington Biochemical Corp., NJ, USA) for 10 min with humidified oxygenation. Cells were then disassociated, washed twice, and plated on the poly-ornithine-coated coverslips at a density of 75,000 cells per cm^2^ in the culture media containing (v/v) 60% Neurobasal-A, 20% Basal Media Eagle, 10% FBS (Atlanta Biologicals, GA, USA) supplemented with 1× B27 and 2 mM GlutaMAX (Thermo Fisher Scientific, MA, USA)^46,47^. The calcium phosphate transfection method was employed to achieve sparse expression and to enable the analysis of a single neuron during the imaging experiments. Following transfection at DIV 3–5, the growth medium was replaced with fresh medium supplemented with an antimitotic agent, ARA-C (Sigma-Aldrich, MO, USA) and glial cell line-derived neurotrophic factor (GDNF) (Millipore, MA, USA). The *Stmn2* shRNA plasmids were purchased from Origene, MD, USA (TF511127), in which the *Stmn2* shRNA expression was driven by a universal U6 promoter with an RFP tag driven by CMV promoter on the same construct.

### Phluorin-based optical assay for endo-/exocytic kinetics

The phluorin-based optical assay was adopted from^46–48^ and performed on DIV12–16 for midbrain cultures. For live cell imaging, cells were mounted on a custom-made laminar-flow stimulation chamber with constant perfusion (at a rate of ~0.2–0.3 mL per min) of a Tyrode’s salt solution containing (in mM) 119 NaCl, 2.5 KCl, 2 CaCl_2_, 2 MgCl_2_, 25 HEPES, 30 glucose, 10 µM 6-cyano-7-nitroquinoxaline-2,3-dione (CNQX), and 50 µM D, L-2-amino-5-phosphonovaleric acid (AP5) and buffered to pH 7.40. All chemicals were purchased from Sigma-Aldrich, except for bafilomycin A1 (1 µM, Calbiochem/Sigma-Aldrich, MO, USA, 196000-1SET). Temperature was clamped at 30.0 °C at the objective throughout the experiment. Field stimulations were delivered at 10 V per cm by A310 Accupulser and A385 stimulus isolator (World Precision Instruments, FL, USA). Images were acquired using a highly sensitive, back-illuminated EM-CCD camera (iXon + Model # DU-897E-BV, Andor Corp., CT, USA). An Olympus IX73 microscope was modified for laser illumination building a solid-state 488 nm OPSL smart laser at 50 mW (used at 10% and output at ~2 mW at the back aperture) into a laser combiner system for millisecond on/off switching and camera blanking control (Andor Corp, NY, USA). An Olympus PLAPON 60XO 1.42 NA objective with a 525/50 m emission filter and 495LP dichroic filters was used for pHluorin fluorescence excitation and collection (Chroma, VT, USA, 49002). Images were sampled at 2 Hz with an Andor Imaging Workstation driven by Andor iQ-CORE-FST (ver 2.x) iQ3.0 software. NH4Cl solution containing (in mM) 50 NH4Cl, 70 NaCl, 2.5 KCl, 2 CaCl_2_, 2 MgCl_2_, 25 HEPES, 30 glucose, 10 µM CNQX, and 50 µM AP5 and buffered to pH 7.40 was used to reveal the total pHluorin expression (total vesicle pool) for normalizing exocytosis. We stimulated the neurons with a short train of 100 pulses (10 Hz 10 s) to measure the endocytosis time constant after the short stimulation (the τ of a fitted one-phase exponential decay after the peak) and the fraction of exocytosis (The peak height of 100 pulses trace divided by the peak height of NH4Cl trace). We also stimulated the neurons with a long train of 300 pulses (10 Hz, 30 s). Bafilomycin (1 µM) was used to block vesicle re-acidification and a train of 1200 pulses (10 Hz, 120 s) was given for measurement of recycling pool and exocytosis time constant (the τ of a fitted one-phase exponential decay since the stimulation starts until the plateau is reached). The peak height difference between 300 pulses and 1200 pulses w/ Bafilomycin traces normalized by the recycling pool refers to the endocytosis fraction during stimulation. Only the neurons co-transfected with the CMV-VMAT2-pHluorin and U6-Stmn2 shRNA-CMV-RFP or U6-scrambled shRNA-CMV-RFP plasmids were recorded for the pHluorin assay. The staging information for each recorded neuron was registered to ensure the re-identification of the same neuron after immunostaining. After recording, the coverslips were fixed and stained with anti-TH antibody to confirm whether the recorded neurons were TH positive.

### In vivo *Stmn2* knockdown mouse model and behavioral tests

Thirty 2-month-old male C57BL/6J mice were purchased from Jackson Laboratory (ME, USA). The IACUC guidelines were strictly followed during animal maintenance and procedure. AAVs carrying either scrambled shRNA or Stmn2-targeting shRNA were packaged by the Boston Children’s Hospital Viral Core. In total, 2 μl of virus with a titer of 1 × 10E13 gc per mL were injected into the right SN (injection coordinates based on Bregma: anterior–posterior: −3.28 mm; medial–lateral: −1.5 mm; dorsal–ventral: −4.1 mm). Behavioral tests were performed during the fourth week after injection.

The locomotor function was tested using a rotarod setting with incremental speed from 4 to 40 RPM. The duration of time an individual mouse remained on the rotarod served as the readout of the experiment. The average duration of three trials was used to represent the performance of the mice.

The overall behavior features were examined by open field test. Individual mice were placed into the 16″ × 16″ animal cage of a Versamax monitor system (Accuscan, NY, USA) in a quiet dark room and allowed to move freely for an hour. The mouse horizontal and vertical movement was monitored and recorded by a grid of 32 infrared beams at ground level and 16 elevated (3″) beams. Then saline was administrated by intraperitoneal (IP) injection, and the mice were placed back into the same cage for 30 min. Finally, amphetamine was administrated by IP injection at a dose of 2.5 mg per kg, and the mice were monitored for another hour in the cage. A series of parameters including total distance, vertical movement, and rotational behavior were analyzed.

### Immunostaining and stereological counting

Cells were fixed by 4% PFA for 10 min and permeabilized in PBS with 0.2% Triton X-100 for 10 min. Then cells were blocked in PBS with 5% BSA for 1 h and incubated with primary antibody diluted in the blocking buffer at 37 °C for 1 h. Cells were then incubated with secondary antibody diluted in the blocking buffer at room temperature for 1 h. The cells were washed four times with PBS between each step, and were incubated in PBS overnight at 4 °C before mounting to reduce background. After behavioral test, mice were anesthetized by ketamine (Vedco Inc. MI, USA, KetaVed) and then perfused with PBS and 4% PFA. The whole brain was taken out and subjected to post fixation in 4% PFA for 24 h and then incubated in 15% and 30% sucrose until fully dehydrated. The brain was cryo-sectioned at 40 μm in thickness using a Leica CM3050s cryostat (Germany). The immunohistochemistry (DAB) was performed according to the manual provided by VECTOR LABORATORIES (Burlingame, CA, USA). For stereology counting, one in every five sections was selected with a random start, and a total of 5–8 brain slices were counted for each mouse using the Stereo Investigator software. Primary antibodies used included Rabbit polyclonal anti-STMN2 (Invitrogen, CA, USA #720178; 1:1000 for western blot and 1:250 for immunostaining); mouse monoclonal anti-beta-actin (Cell Signaling, MA, USA #3700; 1:5000 for western blot); mouse monoclonal anti-TH and rabbit polyclonal anti-TH (Sigma-Aldrich, MO, USA T2928, T8700; 1:1000 for immunostaining); rabbit monoclonal anti-cleaved caspase-3 (Cell Signaling, MA, USA #9579; 1:500 for immunostaining); rat monoclonal anti-DAT (EMD-Millipore, MA, USA MAB369; 1:500 for immunostaining), and chicken polyclonal anti-GFP tag (Life Technology, CA, USA A10262; 1:1000 for immunostaining); rabbit monoclonal anti-Ser129 phosphorylated α-synulein (Abcam, UK, #51253; 1:250 for immunostaining).

### Sample preparation for DA measurement and RNA sequencing

After behavioral test, mice were decapitated and the whole brain was taken out for quick dissection on ice. Striatal samples were sliced and punched to ensure equal amount of tissues. Midbrain samples were carefully isolated, and both samples were separated into the left and right, and were kept at −80 °C until use. Striatal DA content measurement was carried out in neurochemistry core in Vanderbilt University using HPLC. The total RNA were extracted from midbrain samples using Qiagen RNeasy mini kit (Cat No. 74104) and we sent 2.5 μg of each sample to Novogene, CA, USA for pair-end RNA sequencing using 250–300 bp insert cDNA library (rRNA depleted by Ribo-Zero^TM^ Magnetic Kit) on Illumina (CA, USA) HiSeq4000 platform. We applied FastQC (http://www.bioinformatics.babraham.ac.uk/projects/fastqc/) for quality check and then STAR^68^ for alignment followed by edgeR package^69^ for differential gene expression analysis.

### Fractionation of midbrain samples

Right midbrain samples were homogenized in fresh-made brain homogenization buffer (20 mM HEPES pH 7.4, 0.32 M sucrose, 1 mM NaHCO_3_, 2.5 mM CaCl_2_, 1 mM MgCl_2_, protease, and phosphatase inhibitor cocktail (Thermo Fisher Scientific, MA, USA)). The homogenate was centrifuged at 500 rcf for 10 min at 4 °C and supernatant was collected as the starting point. After BCA quantification, the supernatant containing ~250 µg protein was taken from each sample and lysed with 2× lysis buffer (final concentration: 50 mM Tri-HCl pH 7.4, 150 mM NaCl, 1% Triton X, protease, and phosphatase inhibitor). The lysate was incubated on ice for 30 min with gentle shaking. Then the lysate was centrifuged at 16,000 rcf for 30 min at 4 °C. The supernatant was collected as the Triton-soluble fraction. The pellet was washed four times with PBS containing 1% Triton X and centrifuged at 16,000 rcf for 10 min at 4 °C. Then the pellet was resuspended and incubated in 50 µl PBS containing 2% SDS, 1%Triton X, and protease and phosphatase inhibitor at 60 °C for 1 h. The samples were centrifuged again at 16,000 rcf for 30 min at 4 °C. The supernatant was collected as the Triton-insoluble fraction. For Triton-soluble fraction, we quantified the concentration by BCA assay and loaded 25 µg protein; for Triton-insoluble fraction, we added 6x sample buffer and load 25 µl of samples.

### Western blot

Sample concentration was determined by BCA (Pierce, MA, USA). Then samples were boiled in 3× sample buffer at 95 °C for 10 min and briefly centrifuged. In all, 30 µg protein samples were loaded into 4–12% gradient gels (Invitrogen, CA, USA) and run at 120 V in 1× MOPS running buffer. Transfer was done at 4 °C in transfer buffer with 10% methanol for 2 h at 90 V. The membrane was blocked in LI-COR blocking buffer for 1 h and then overnight incubation with primary antibody at 4 °C. Then the membrane was washed three times in TBST buffer and incubated in fluorescence-conjugated secondary antibody (1:10000, LI-COR, Germany) for 1 h. The membrane was washed three times in TBST buffer with 0.01% SDS followed by imaging in LI-COR imaging system.

### Quantification and statistical analysis

The data analysis was performed by Bioconductor R 3.4.3. Images were processed by ImageJ. Data were represented as mean ± SEM or mean ± 95% confidence interval (CI) as specified. Student’s *t* test was used for between two-group comparisons. ANOVA and Tukey post hoc comparison were applied in multi-group/factorial design. Fisher’s exact test was used to examine whether two sets were significantly overlapped/enriched. BH-corrected *P* < 0.05 was considered as statistically significant unless stated otherwise. The details, including the *N* number, the statistic tests used and the *p-*values, are indicated in each figure and figure legend.

### Reporting summary

Further information on research design is available in the Nature Research Reporting Summary linked to this article.

## Supplementary information

Supplementary Information

Dataset 1

Dataset 2

Dataset 3

Dataset 4

Dataset 5

Dataset 6

Dataset 7

Dataset 8

Dataset 9

Dataset 10

Dataset 11

Dataset 12

Description of Additional Supplementary Files

Reporting Summary

Source Data

## Data Availability

The human SN expression profiles are downloaded from the gene expression omnibus (GEO) (https://www.ncbi.nlm.nih.gov/geo/) with accession number GSE8397, GSE7621, GSE24378, GSE20292, GSE20141, GSE20163, GSE20164, GSE49036. RNA sequencing data from *Stmn2*-knockdown mouse brains are deposited to GEO with accession number GSE114840. The source data underlying Figs. 3a–c, e, 5b–f, 6a, c, 7b, c, f, h and supplementary Figs. 4, 5c, e, 6a, b, d–g are provided as a Source Data file. All the other data are contained in the article and its supplementary information or available upon request.

## Code availability

The R packages MEGENA and Metafor are available from https://cran.r-project.org/ and the code for Bayesian network inference can be found through RIMBANet (https://icahn.mssm.edu/research/genomics/about/resources).
