# Supplementary material for: The landscape of multiscale transcriptomic networks and key regulators in Parkinson’s disease
Source: Nat Commun. 2019 Nov 20;10:5234. doi: 10.1038/s41467-019-13144-y (PMC6868244; doi:10.1038/s41467-019-13144-y)
Supplement: Supplementary file 1 — Supplementary Information [file 41467_2019_13144_MOESM1_ESM.pdf]

The Landscape of Multiscale Transcriptomic Networks and Key Regulators in Parkinson's Disease

Wang *et al.*

Supplementary Note 1

We evaluated the conservation between the MEGENA modules and those from the traditional Weighted Gene Co-expression Network Analysis (WGCNA). A total of 24 WGCNA modules with at least 20 genes were identified (Supplementary Fig. 3a and 3b). We found that 75 of the 90 MEGENA modules were significantly enriched in the WGCNA modules while all the 24 WGCNA modules were significantly enriched in the MEGENA modules at cFET $p < 0.05$ (Supplementary Fig. 3c and Supplementary Data 7). Therefore, 15 modules were unique to the MEGENA network. For examples, M576, which was significantly associated with regulation of action potential, was only identified by MEGENA. More importantly, MEGENA provides more attractive features far beyond the simple clustering analysis offered by WGCNA such as identification of the hierarchical structures of modules, sparsification of dense correlations and determination of key regulators. Such features enable generation of explicit network models and thus provide more biological insights than simple presentation of gene clusters. As shown in Supplementary Fig. 3c, many WGCNA modules were dissected into high resolution MEGENA modules with clear network topology. For example, the turquoise and blue modules in WGCNA were significantly enriched in multiple branches of MEGENA modules, which were associated with distinct biological functions (Supplementary Fig. 3c and Supplementary Data 5). Additionally, the cell type specificity analysis showed that the oligodendrocyte-enriched M12 branch in MEGENA (Supplementary Fig. 3e) was not identified by WGCNA (Supplementary Fig. 3d). Taken together, MEGENA provided more coherent and functionally relevant modules with high resolution topological network structures and clearly defined key regulators for downstream analyses.

Supplementary Figures

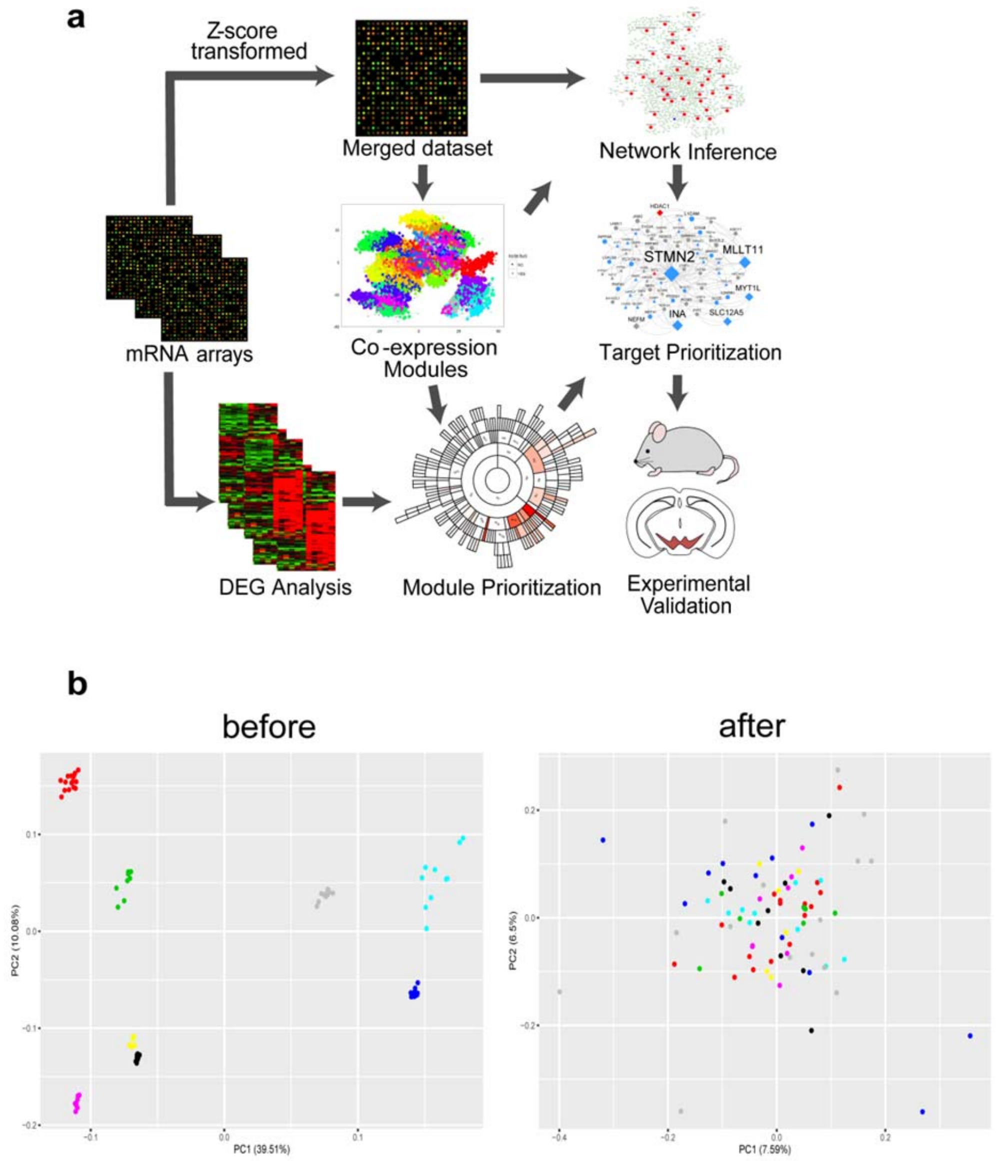

Supplementary Figure 1. The architecture of the integrative network biology approach for PD. (**a**) Microarray based gene expression datasets from the healthy and PD postmortem human brains were collected from the GEO database. From the microarray RNA expression data, we first identified differentially expressed gene signatures between the controls and PD patients by meta-analysis. Then each dataset went through Z-score transformation followed by correction for

covariates such as gender and then merged into a combined dataset including both PD cases and controls. The combined data from the PD cases were used for the co-expression network construction and causal network inference. The modules identified in the co-expression network of PD were intersected with the DEG signatures of PD and other PD related gene signatures such as PD GWAS genes and the known PD pathway genes for prioritization. The cell-type specific marker signatures were also used for characterizing cell type specificity of the modules. We also constructed a Bayesian causal network for each module. Key regulators for each module were identified by both the respective MEGENA subnetwork and Bayesian network. Finally, the key regulators of the module most associated were prioritized for experimental validation. (**b**) Principle component analysis (PCA) plot before and after z-score transformation for the PD datasets. Colors indicate samples from different studies.

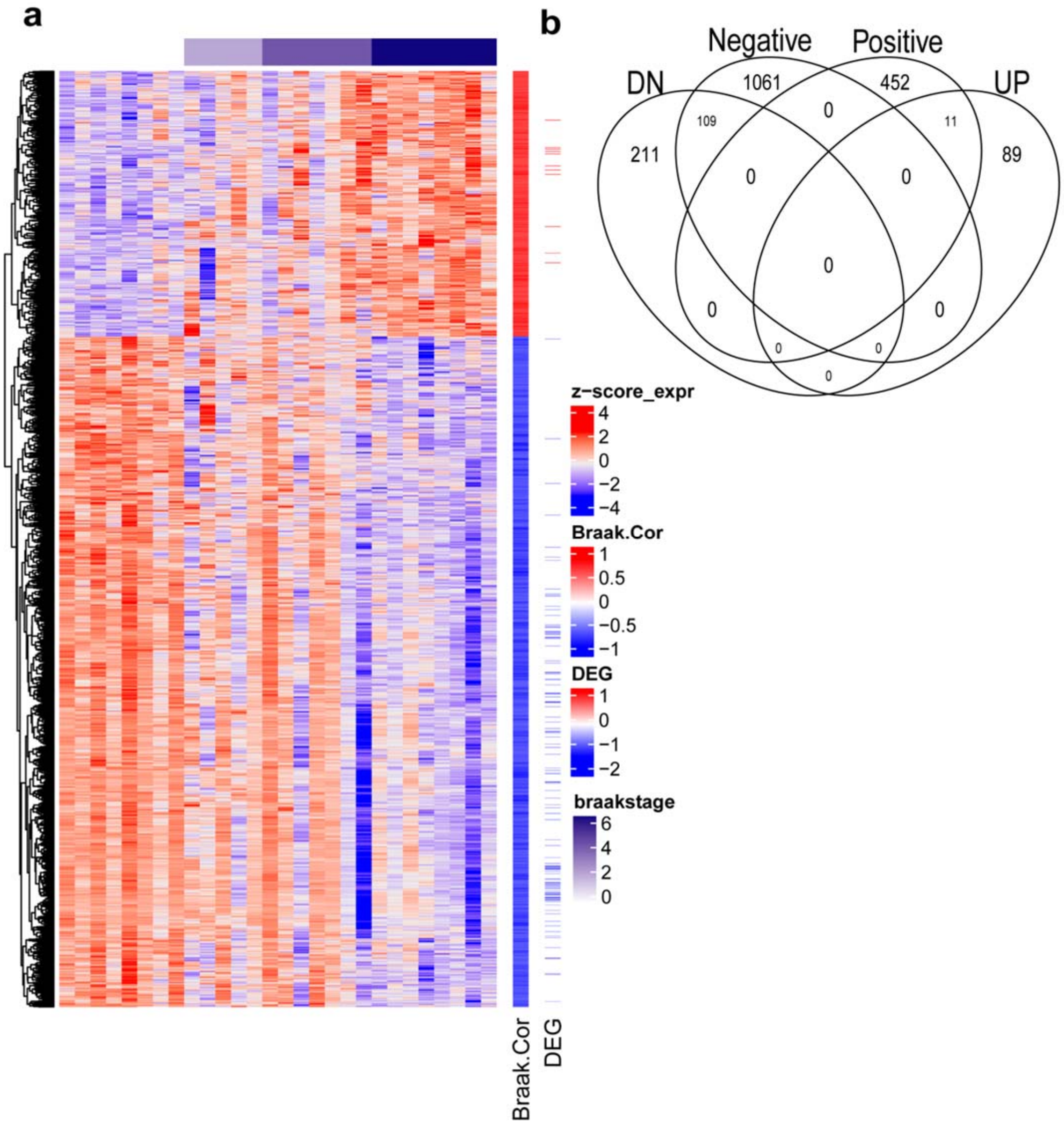

Supplementary Figure 2. Comparison of the DEGs and the BCGs in the SN of the PD patients. (**a**) Gene expression of the BCGs in the dataset GSE49036 with Braak stage represented on the top of the heatmap and the DEGs in the meta-analysis of the rest 7 datasets on the side. (**b**) Venn diagram of the BCGs and the DEGs mentioned in (**a**).

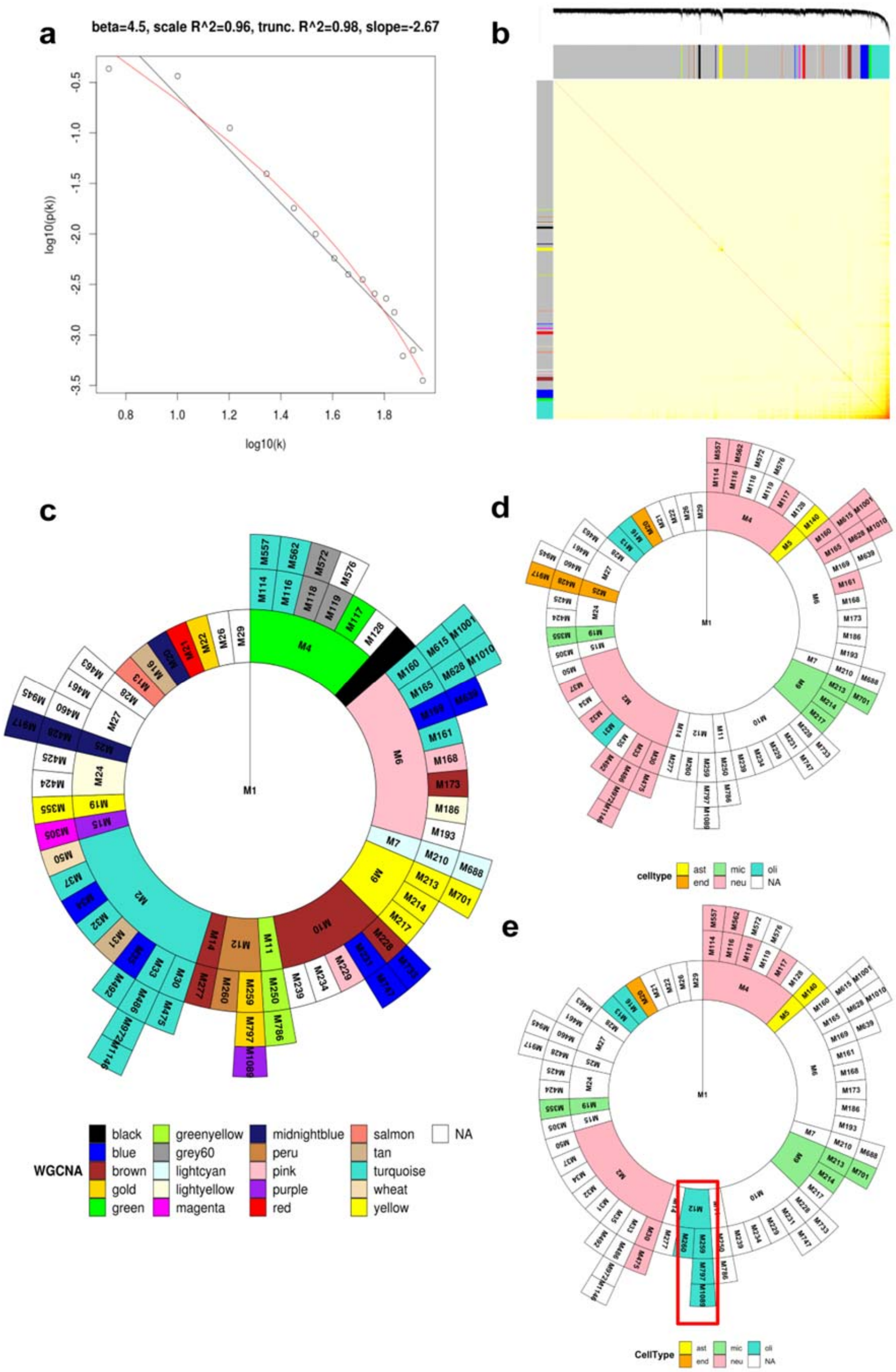

Supplementary Figure 3. Comparison of the modules from MEGENA and those from WGCNA. (a) Parameters chosen to fit the scale free topology in WGCNA. (**b**) The heatmap of the modules identified by WGCNA. (**c**) Conservation of the modules identified by WGCNA and

those by MEGENA. The sunburst plot showed the hierarchical organization of the MEGENA modules and their enrichment for the WCGNA modules which were represented by different color names. A MEGENA module (Mxx) in a color (Y) other than white indicates a significant conservation between Mxx and the WGCNA module represented by the color Y. (**d**) Projection of WGCNA module enrichment with cell type markers onto MEGENA sunburst plot. Cell type specificity of each WGCNA module was first determined by FET test with cell type markers and then matched to its overlapping MEGENA modules identified in (**c**), e.g, the microglia-enriched yellow module in WGCNA was significantly overlapped with the M9 and M19 branches in MEGENA. Therefore, these two branches were colored in lightgreen for microglia. (**e**) MEGENA module enrichment for cell type specific marker signatures. Red box highlighted the additional information MEGENA provided compared to WGCNA.

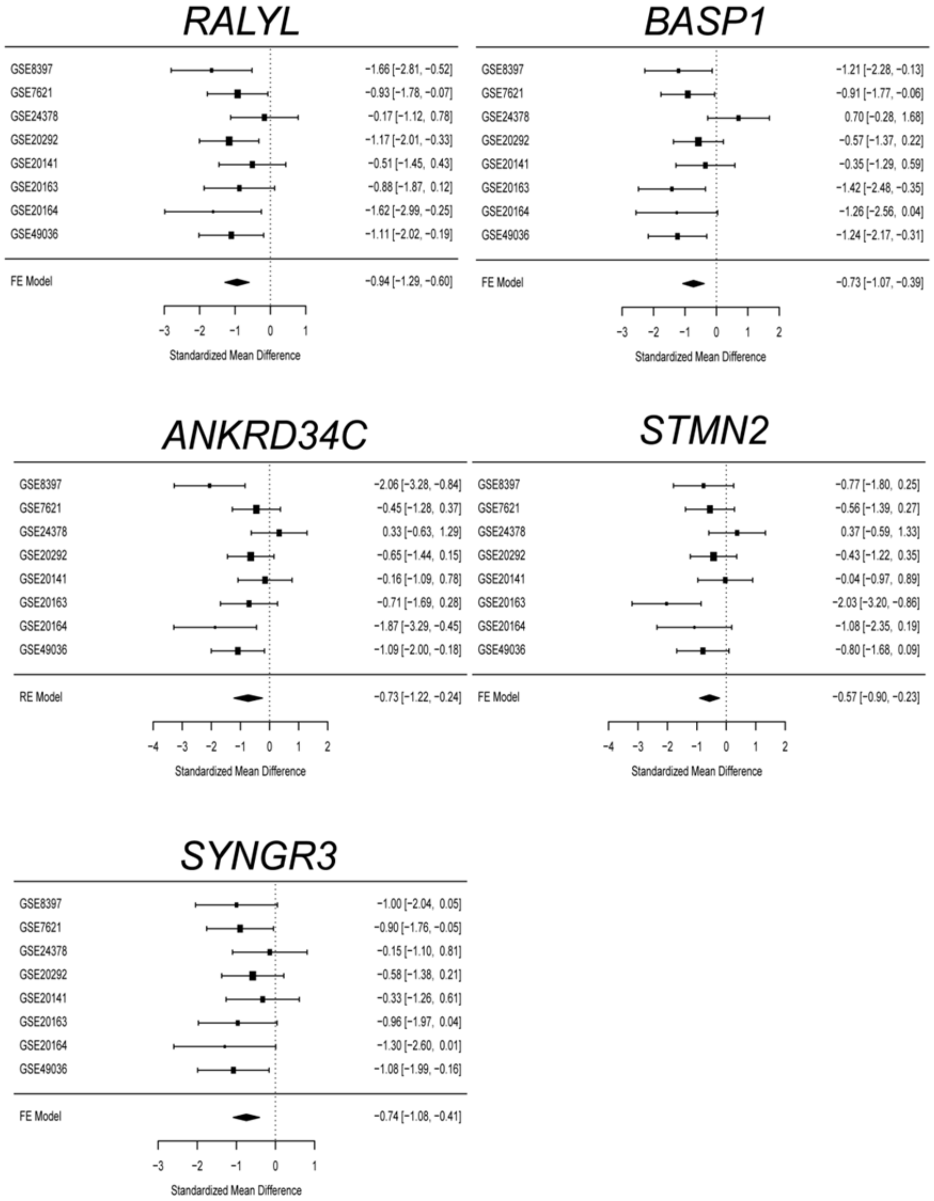

Supplementary Figure 4. The meta-analysis of the top 5 key regulators in M4. Standardized mean differences of the gene expressions of the top 5 key regulators in the SN between the postmortem PD and control brains in 8 human PD studies were shown here. A fixed effect model was applied to *RALYL, BASP1*, *STMN2* and *SYNGR3* based on heterogeneity test while a random effect model was applied to *ANKRD34C*. All data are present as Mean SMD [95% CI].

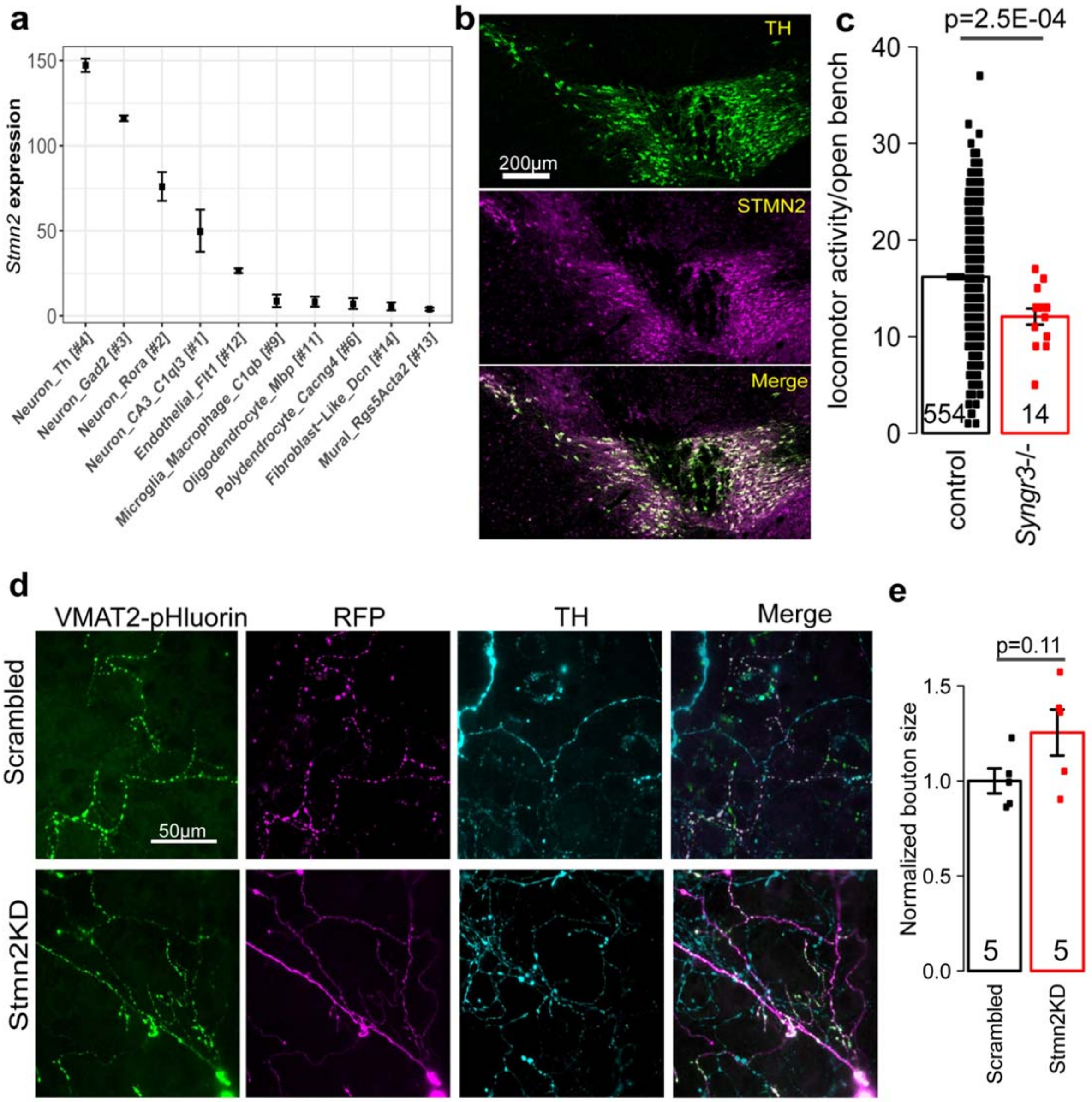

Supplementary Figure 5. Prioritization of top candidates for experimental validation. (a) *Stmn2* expression was found enriched in TH+ neurons (www.dropviz.org). Data are present as Mean±95% CI. (**b**) STMN2 expression was found enriched in TH+ neurons in the brain slices in 3-month-old wildtype C57BL/6J mice. (**c**) Locomotor activity measured in open bench assay is impaired in *Syngr*3$^{-/-}$ mice. N=554 for control, N=14 for *Syngr3*$^{-/-}$; two-sided Student's *t*-test for two group comparison, t=4.7332, df=15.259, p=2.5E-04. (**d**) Immunostaining of VMAT2-

pHluorin, RFP tag of *Stmn2*-shRNA plasmid and TH of recorded non-TH neurons treated with scrambled or *Stmn2*-shRNA. (**e**) Quantification of bouton sizes in scrambled and *Stmn2*-shRNA treated non-TH neurons. Two-sided Student's *t*-test for two group comparison. t=1.8464, df=8, p-value=0.11. Data in (**c**) and (**e**) are present as Mean±SEM.

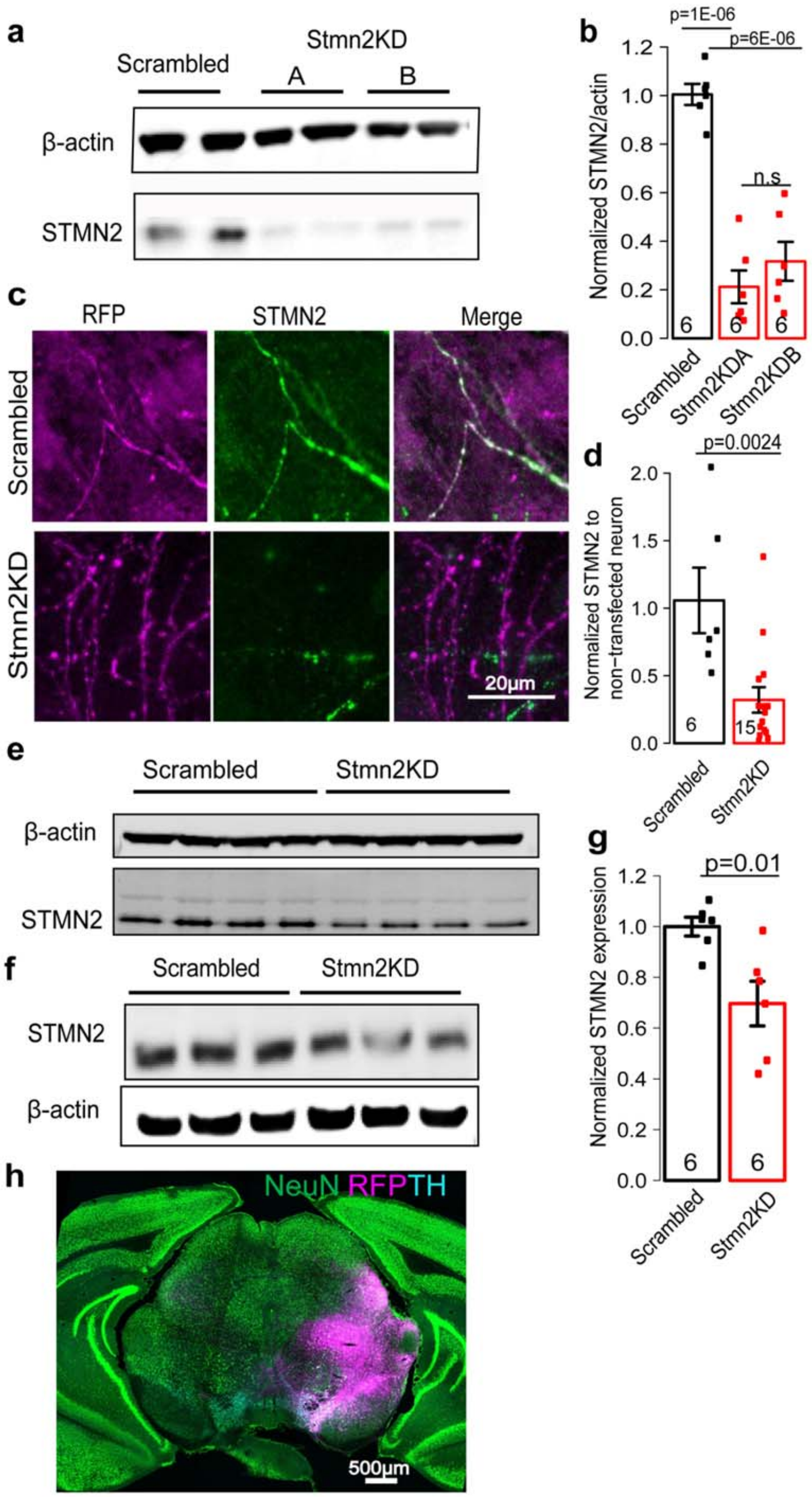

Supplementary Figure 6. Knockdown efficiency of *Stmn2*-shRNA construct and AAV. (**a**) The efficiency of two *Stmn2*-shRNA constructs was tested by western blot in N2A cells. Two

biological replicates in each experiment and three independent experiments were performed. (**b**) The densitometry quantification of Stmn2 shRNA knockdown efficiency. One-way ANOVA and TukeyHSD test were used to compare the differences between groups. F=43.27, df=2, p=5.9E-07; Stmn2KDA-Scrambled, p=1E-06; Stmn2KDB-Scrambled, p=6E-06. (**c**) Immunostaining of STMN2 in the scrambled-treated and *Stmn2* knockdown midbrain cultures. Construct B was used in all following experiments and the knockdown efficiency was consistent with what was observed in N2A cells. (**d**) The densitometry quantification of *Stmn2* shRNA knockdown efficiency in cultured midbrain neurons. Two-sided Student's *t*-test, t= 3.4909, df = 19, p-value = 0.0024. (**e**) The knockdown efficiency of *Stmn2*-shRNA AAV at 72 hours post-infection in N2A cells. (**f**) Western blot of the midbrain tissues isolated from mice received either scrambled AAV or *Stmn2* knockdown AAV. (**g**) Densitometry quantification of STMN2/actin in the control and *Stmn2* knockdown group. N=6 in each group, two-sided Student's *t*-test. t =3.1763, df = 10, p-value = 0.009881. (**h**) Viral spreading after AAV injection in SN. All data are present as Mean±SEM.

Supplementary Tables

Supplementary Table 1. Human postmortem brain studies of PD in public domain

GEO ID	Brain Region	Control	PD	Platform	Pre-Processed	Note
GSE8397	lateral substantia nigra	7	9	Affymetrix Human Genome U133A Array	RMA	
GSE7621	substantia nigra	9	16	Affymetrix Human Genome U133 Plus 2.0 Array	MAS5	
GSE24378	substantia nigra	9	8	Affymetrix Human X3P Array	MAS5	
GSE20292	substantia nigra	15	11	Affymetrix Human Genome U133A Array	MAS5	3 control samples were removed as outliers
GSE20141	substantia nigra	8	10	Affymetrix Human Genome U133 Plus 2.0 Array	MAS5	
GSE20163	substantia nigra	9	8	Affymetrix Human Genome U133A Array	MAS5	
GSE20164	substantia nigra	5	6	Affymetrix Human Genome U133A Array	MAS5	
GSE49036	substantia nigra	8	15	Affymetrix Human Genome U133 Plus 2.0 Array	GC-RMA	5 iLBD samples were excluded for DEG and network analysis
	total	70	83			

Supplementary Table 2. Module ranking

module	Score.rank	CellType	CellType.adj.p	ALL	DN	UP	GWAS.Catalog	hsa05012
M4	25.1023531	neu	6.17E-28	7.90E-26	2.69E-29	1	1	1
M116	22.66096449	neu	3.00E-18	2.18E-23	7.35E-23	1	1	1
M2	18.66254901	neu	2.93E-06	1.97E-15	1.16E-24	1	1	0.00011
M30	18.58976303	neu	1.15E-22	2.57E-19	1.08E-22	1	1	1
M562	16.14588077	neu	3.79E-11	7.15E-17	2.72E-15	0.036445	1	1
M1001	13.94404107	NA	1	1.14E-14	2.10E-15	1	1	1
M475	13.57204579	neu	7.86E-18	2.68E-14	3.52E-17	1	1	1
M114	12.43914742	neu	2.44E-11	3.64E-13	1.25E-12	1	1	1
M557	12.43790895	neu	4.32E-10	3.65E-13	1.45E-13	1	1	1
M160	12.36364902	NA	1	4.33E-13	6.38E-15	1	1	1
M615	11.26171502	NA	1	5.47E-12	2.70E-13	1	1	1
M117	11.17682777	neu	0.026989276	6.66E-12	9.88E-15	1	1	1
M32	10.82206669	NA	1	3.26E-08	6.77E-10	1	1	0.000462
M628	8.540822829	NA	1	1.02E-05	1.95E-07	1	1	0.000281
M165	5.955588198	NA	1	6.48E-05	4.48E-07	1	1	0.017099
M1010	5.052757604	NA	1	0.000518	8.61E-05	1	1	0.017099
M37	3.632234436	NA	1	0.000233	3.39E-06	1	1	1
M1146	3.285726316	NA	1	0.000518	1.67E-05	1	1	1
M733	2.650169463	NA	1	0.002238	0.001671	1	1	1
M486	2.573232849	NA	1	0.002672	0.000255	1	1	1
M688	2.246880706	NA	1	0.005664	0.000945	1	1	1
M972	2.246880706	NA	1	0.005664	0.00026	1	1	1
M31	2.167128306	NA	1	0.006806	0.008668	1	1	1
M492	2.093689411	NA	1	0.00806	0.000528	1	1	1
M33	2.085631265	NA	1	0.00821	0.000305	1	1	1
M35	2.071964352	NA	1	0.008473	0.001242	1	1	1
M161	1.765810921	NA	1	1	1	1	1	0.017147
M34	1.765810921	NA	1	1	1	1	1	0.017147
M16	1.353818955	oli	4.12E-19	0.044277	1	2.98E-10	1	1
M9	0	mic	2.31E-69	1	1	1	1	1
M213	0	mic	3.59E-41	1	1	1	1	1
M5	0	ast	4.27E-37	1	1	1	1	1
M701	0	mic	1.11E-31	1	1	1	1	1
M20	0	end	1.31E-28	1	1	1	1	1
M140	0	ast	2.05E-24	1	1	1	1	1
M13	0	oli	5.10E-22	1	1	0.000518	1	1
M214	0	mic	7.79E-15	1	1	1	1	1
M19	0	mic	9.62E-13	1	1	1	1	1
M355	0	mic	3.16E-09	1	1	1	1	1
M12	0	oli	2.05E-07	1	1	1	1	1
M259	0	oli	1.07E-06	1	1	1	1	1
M797	0	oli	1.25E-06	1	1	1	1	1
M1089	0	oli	1.60E-06	1	1	1	1	1
M260	0	oli	0.00951514	1	1	1	1	1
M118	0	neu	0.019373572	1	1	1	1	1
M10	0	NA	1	1	1	1	1	1
M11	0	NA	1	1	1	0.006507	1	1
M119	0	NA	1	1	1	1	1	1

M128	0	NA	1	1	1	1	1	1
M14	0	NA	1	1	1	1	1	1
M15	0	NA	1	1	1	1	1	1
M168	0	NA	1	1	1	1	1	1
M169	0	NA	1	1	1	1	1	1
M173	0	NA	1	1	1	1	1	1
M186	0	NA	1	1	1	1	1	1
M193	0	NA	1	1	1	1	1	1
M21	0	NA	1	1	1	1	1	1
M210	0	NA	1	1	0.019243	1	1	1
M217	0	NA	1	1	1	1	1	1
M22	0	NA	1	1	1	1	1	1
M228	0	NA	1	1	0.017528	1	1	1
M229	0	NA	1	1	1	1	1	1
M231	0	NA	1	1	1	1	1	1
M234	0	NA	1	1	1	1	1	1
M239	0	NA	1	1	1	1	1	1
M24	0	NA	1	1	1	1	1	1
M25	0	NA	1	1	1	1	1	1
M250	0	NA	1	1	1	0.002644	1	1
M26	0	NA	1	1	1	1	1	1
M27	0	NA	1	1	1	1	1	1
M277	0	NA	1	1	1	1	1	1
M28	0	NA	1	1	1	1	1	1
M29	0	NA	1	1	1	1	1	1
M305	0	NA	1	1	1	1	1	1
M424	0	NA	1	1	1	1	1	1
M425	0	NA	1	1	1	1	1	1
M428	0	NA	1	1	1	1	1	1
M460	0	NA	1	1	1	1	1	1
M461	0	NA	1	1	1	1	1	1
M463	0	NA	1	1	1	1	1	1
M50	0	NA	1	1	1	1	1	1
M572	0	NA	1	1	1	1	1	1
M576	0	NA	1	1	1	1	1	1
M6	0	NA	1	1	0.001303	1	1	1
M639	0	NA	1	1	1	1	1	1
M7	0	NA	1	1	1	1	1	1
M747	0	NA	1	1	1	1	1	1
M786	0	NA	1	1	1	4.24E-05	1	1
M917	0	NA	1	1	1	1	1	1
M945	0	NA	1	1	1	1	1	1

Supplementary Table 3. M4 hub ranking

id	MEGENA	BN	comb.rank.score	DEG
RALYL	3	1	0.002403	DN
BASP1	2	5	0.00226	DN
ANKRD34C	4	3	0.002222	DN
STMN2	5	6	0.001998	DN
SYNGR3	1	11	0.001989	DN
FGF13	8	7	0.001746	DN
DDC	11	2	0.001743	DN
NELL2	6	16	0.001447	DN
NUDT11	7	18	0.001302	DN
SLC6A3	17	9	0.001083	DN
SYN1	14	24	0.000758	NA
FBXW4	12	27	0.00072	UP
RUSC1	19	19	0.000698	NA
CNTNAP1	18	30	0.000441	NA
MAST2	20	31	0.000362	NA
PIP5K1C	29	45	8.61E-06	NA

Supplementary Table 4. Cell type specific expression of top regulators in adult mouse brain (www.dropviz.org)

Region	Class	Cluster	Ralyl Expr	Ralyl P-Val	Basp1 Expr	Basp1 P-Val	Ankrd34c Expr	Ankrd34c P-Val	Stmn2 Expr	Stmn2 P-Val	Syngr3 Expr	Syngr3 P-Val
Substantia Nigra	Neuron	Neuron_CA3_C1ql3 [#1]	2.56	1.2E-08	4.95	4.6E-111	0	1	3.61	0.15	2.64	0.11
Substantia Nigra	Neuron	Neuron_Gad2 [#3]	2.48	0	4.62	0	0.69	1.9E-85	4.56	0	2.89	0
Substantia Nigra	Neuron	Neuron_Th [#4]	2.20	6.3E-41	4.62	0	0.69	0.007	4.80	0	3.47	0
Substantia Nigra	Neuron	Neuron_Rora [#2]	1.39	0.37	4.45	1.1E-83	1.79	2.0E-19	4.16	7.1E-29	2.48	7.1E-07
Substantia Nigra	Polydendrocyte	Polydendrocyte_Cacng4 [#6]	1.39	0.43	4.50	1.2E-48	0	0.64	2.08	7.3E-18	1.79	0.35
Substantia Nigra	Microglia_ Macrophage	Microglia_Macrophage_C1qb [#9]	0.69	0.04	3.93	0.001	0	0.63	2.08	2.9E-14	0.69	0.001
Substantia Nigra	Oligodendrocyte	Oligodendrocyte_Tfr [#10]	0	0	1.61	0	0	2.6E-73	1.61	0	0.69	0
Substantia Nigra	Oligodendrocyte	Oligodendrocyte_Mbp [#11]	0	1.1E-05	2.20	4.3E-20	0	0.43	2.08	1.5E-23	0.69	2.6E-06
Substantia Nigra	Endothelial	Endothelial_Flt1 [#12]	0	6.0E-75	1.39	0	0	1.9E-11	3.00	4.2E-65	0	6.0E-141
Substantia Nigra	Mural	Mural_Rgs5Acta2 [#13]	0	1.2E-22	1.39	2.0E-127	0	0.006	1.61	3.0E-136	0	1.1E-43
Substantia Nigra	Fibroblast-Like	Fibroblast-Like_Dcn [#14]	0	4.9E-07	1.95	2.6E-26	0	1	1.79	1.5E-28	1.10	4.3E-06
Substantia Nigra	Polydendrocyte	Polydendrocyte_Tnr [#5]	0	4.0E-18	2.64	5.1E-68	0	0.004	1.39	1.8E-112	0.69	1.5E-29
Substantia Nigra	Astrocyte	Astrocyte_Gja1 [#7]	0	5.4E-38	2.89	2.9E-60	0	0.002	1.39	1.6E-231	0.69	5.3E-66
Substantia Nigra	Ependyma	Ependyma [#8]	0	0.73	2.20	0.001	0	1	1.10	4.7E-06	0	0.041
