# Supplementary material for: The landscape of multiscale transcriptomic networks and key regulators in Parkinson’s disease
Source: Nat Commun. 2019 Nov 20;10:5234. doi: 10.1038/s41467-019-13144-y (PMC6868244; doi:10.1038/s41467-019-13144-y)
Supplement: Supplementary file 14 — Description of Additional Supplementary Files [file 41467_2019_13144_MOESM14_ESM.pdf]

Title: Supplementary Data 1.
Description: The DEGs identified by the meta-analysis of the human PD datasets.

Title: Supplementary Data 2.
Description: The DEGs identified by the meta-analysis of the human PD datasets without GSE49036.

Title: Supplementary Data 3.
Description: The genes correlated with Braak stage in the dataset GSE49036.

Title: Supplementary Data 4.
Description: The gene ontology terms enriched in the PD DEGs.

Title: Supplementary Data 5.
Description: Annotation of the MEGENA modules by enriched GO terms.

Title: Supplementary Data 6.
Description: The top 500 marker genes for each of the 6 major brain cell types.

Title: Supplementary Data 7.
Description: Functional annotation of the key regulators of the module M4 by their 2- layer network neighborhoods.

Title: Supplementary Data 8.
Description: Functional annotation of the PD GWAS genes by their 2-layer network neighborhoods.

Title: Supplementary Data 9.
Description: Enrichment of the PD DEGs in the 2-layer network neighborhoods of the PD GWAS genes.

Title: Supplementary Data 10.
Description: The genes in response to Stmn2 knockdown in the mouse SN.

Title: Supplementary Data 11.
Description: The STMN2-correlated genes in the combined dataset of PD patients.

Title: Supplementary Data 12.
Description: Conservation between the MEGENA and WGCNA modules.
