# Supplementary material for: The landscape of multiscale transcriptomic networks and key regulators in Parkinson’s disease
Source: Nat Commun. 2019 Nov 20;10:5234. doi: 10.1038/s41467-019-13144-y (PMC6868244; doi:10.1038/s41467-019-13144-y)
Supplement: Supplementary file 15 — Reporting Summary [file 41467_2019_13144_MOESM15_ESM.pdf]

nature research

Corresponding author(s): NCOMMS-19-01999C

Last updated by author(s): Oct 8, 2019

Reporting Summary

Nature Research wishes to improve the reproducibility of the work that we publish. This form provides structure for consistency and transparency in reporting. For further information on Nature Research policies, see Authors & Referees and the Editorial Policy Checklist .

Statistics

For all statistical analyses, confirm that the following items are present in the figure legend, table legend, main text, or Methods section.

n/a	Confirmed	
☐	☒	The exact sample size (*n*) for each experimental group/condition, given as a discrete number and unit of measurement
☐	☒	A statement on whether measurements were taken from distinct samples or whether the same sample was measured repeatedly
☐	☒	The statistical test(s) used AND whether they are one- or two-sided<br>*Only common tests should be described solely by name; describe more complex techniques in the Methods section.*
☒	☐	A description of all covariates tested
☐	☒	A description of any assumptions or corrections, such as tests of normality and adjustment for multiple comparisons
☐	☒	A full description of the statistical parameters including central tendency (e.g. means) or other basic estimates (e.g. regression coefficient) AND variation (e.g. standard deviation) or associated estimates of uncertainty (e.g. confidence intervals)
☐	☒	For null hypothesis testing, the test statistic (e.g. *F*, *t*, *r*) with confidence intervals, effect sizes, degrees of freedom and *P* value noted<br>*Give P values as exact values whenever suitable.*
☐	☒	For Bayesian analysis, information on the choice of priors and Markov chain Monte Carlo settings
☐	☒	For hierarchical and complex designs, identification of the appropriate level for tests and full reporting of outcomes
☐	☒	Estimates of effect sizes (e.g. Cohen's *d*, Pearson's *r*), indicating how they were calculated

Our web collection on statistics for biologists contains articles on many of the points above.

Software and code

Policy information about availability of computer code

Data collection	Gene expression profiles were downloaded from GEO database (https://www.ncbi.nlm.nih.gov/geo/) and were listed in Supplementary Table 1. Accession number and links were provided in Data Availability Statement
Data analysis	Data analysis was performed using R 3.3.2-3.4.3. Main packages used including MEGENA and RIMBANET, which were already published. Please see Code Availability Statement.

For manuscripts utilizing custom algorithms or software that are central to the research but not yet described in published literature, software must be made available to editors/reviewers. We strongly encourage code deposition in a community repository (e.g. GitHub). See the Nature Research guidelines for submitting code & software for further information.

Data

Policy information about availability of data

All manuscripts must include a data availability statement. This statement should provide the following information, where applicable:

- Accession codes, unique identifiers, or web links for publicly available datasets
- A list of figures that have associated raw data
- A description of any restrictions on data availability

The human SN expression profiles are downloaded from the gene expression omnibus (GEO) (https://www.ncbi.nlm.nih.gov/geo/) with accession number GSE8397 [https://www.ncbi.nlm.nih.gov/geo/query/acc.cgi?acc=GSE8397], GSE7621 [https://www.ncbi.nlm.nih.gov/geo/query/acc.cgi?acc=GSE7621], GSE24378 [https://www.ncbi.nlm.nih.gov/geo/query/acc.cgi?acc=GSE24378], GSE20292 [https://www.ncbi.nlm.nih.gov/geo/query/acc.cgi?acc=GSE20292], GSE20141 [https://www.ncbi.nlm.nih.gov/geo/query/acc.cgi?acc=GSE20141], GSE20163 [https://www.ncbi.nlm.nih.gov/geo/query/acc.cgi?acc=GSE20163], GSE20164 [https://www.ncbi.nlm.nih.gov/geo/query/acc.cgi?acc=GSE20164], GSE49036 [https://www.ncbi.nlm.nih.gov/geo/query/acc.cgi?acc=GSE49036]. RNA-seq sequencing data from Stmn2-knockdown mouse brains are deposited to GEO with accession number GSE114840. The source data underlying Fig.3A-C, 3E, Fig. 5B-F, Fig. 6A, 6C, Fig. 7B, 7C, 7F, 7H and supplementary Figure 4, 5C, 5E, 6A, 6B, 6D-G are provided as a Source Data file. All the other data are contained in the article and its supplementary information or available upon request.

Field-specific reporting

Please select the one below that is the best fit for your research. If you are not sure, read the appropriate sections before making your selection.

- [x] Life sciences
- [ ] Behavioural & social sciences
- [ ] Ecological, evolutionary & environmental sciences

For a reference copy of the document with all sections, see nature.com/documents/nr-reporting-summary-flat.pdf

Life sciences study design

All studies must disclose on these points even when the disclosure is negative.

Sample size	No sample size calculation was performed. Sample size was chosen empirically.
Data exclusions	One sample from RNA sequencing was excluded because of low RNA integrity.<br>We excluded the neurons dying during pHluorin recording for endo- and exo-cytosis.<br>We also exclude data points defined as outliers in boxplot in R
Replication	We tried different methods to reproduce our findings. For example, we used both western blot and RNAseq to confirm the knockdown efficiency of Stmn2 in vivo. We have biological replicates for each experiments.
Randomization	Mice were randomized by random number generator into two groups.
Blinding	Mice were randomized by random number generator into two groups.

Reporting for specific materials, systems and methods

We require information from authors about some types of materials, experimental systems and methods used in many studies. Here, indicate whether each material, system or method listed is relevant to your study. If you are not sure if a list item applies to your research, read the appropriate section before selecting a response.

Materials & experimental systems

n/a	Involved in the study	
☐	☒	Antibodies
☐	☒	Eukaryotic cell lines
☒	☐	Palaeontology
☐	☒	Animals and other organisms
☒	☐	Human research participants
☒	☐	Clinical data

Methods

n/a	Involved in the study	
☒	☐	ChIP-seq
☒	☐	Flow cytometry
☒	☐	MRI-based neuroimaging

Antibodies

Antibodies used	Primary antibodies used included Rabbit polyclonal anti-STMN2 (Invitrogen, CA, USA #720178; 1:1000 for western blot and 1:250 for immunostaining); Mouse monoclonal anti-beta-actin (Cell Signaling, MA, USA #3700; 1:5000 for western blot); Mouse monoclonal anti-TH and rabbit polyclonal anti-TH (Sigma-Aldrich, MO, USA T2928, T8700; 1:1000 for immunostaining); Rabbit monoclonal anti-cleaved caspase 3 (Cell Signaling, MA, USA #9579; 1:500 for immunostaining); Rat monoclonal anti-DAT (EMD-Millipore, MA, USA MAB369; 1:500 for immunostaining) and Chicken polyclonal anti-GFP tag (Life Technology, CA, USA A10262; 1:1000 for immunostaining); Rabbit monoclonal anti-Ser129 phosphorylated α-synulein (Abcam, UK, #51253; 1:250 for immunostaining).
Validation	The primary antibodies used are validated by the manufacturers with the validation and citation information provided on the corresponding websites.

Eukaryotic cell lines

Policy information about cell lines

Cell line source(s)	mouse neuroblastoma N2A cell line was orginally from ATCC CCL-131
Authentication	The cell line was authenticated by ATCC

Mycoplasma contamination	The cell line was not tested for mycoplasma contamination
Commonly misidentified lines (See ICLAC register)	*Name any commonly misidentified cell lines used in the study and provide a rationale for their use.*

Animals and other organisms

Policy information about studies involving animals; ARRIVE guidelines recommended for reporting animal research

Laboratory animals	2 month-old C57BL/6J male mice were purchased from Jackson Laboratory, ME, USA
Wild animals	The study did not involve wild animals
Field-collected samples	The study did not involve filed-collected samples
Ethics oversight	Institutional Animal Care and Use Committee (IACUC) in Icahn School of Medicine at Mount Sinai

Note that full information on the approval of the study protocol must also be provided in the manuscript.
